# Social Contexts Requiring Adjudication Self- and Peer-Interest Differentially Alter Risk Preferences Across Adolescence

**DOI:** 10.1162/opmi_a_00201

**Published:** 2025-04-22

**Authors:** Yelina Yiyi Chen, Gail M. Rosenbaum, Haoxue Fan, John C. Flournoy, Tianxiang Li, Laura Cegarra, Deanna A. Youssoufian, Melanie J. Grad-Freilich, Laurel E. Kordyban, Patrick Mair, Leah H. Somerville

**Affiliations:** Department of Psychology, Harvard University; Department of Psychology, New York University; Center for Brain Science, Harvard University

**Keywords:** adolescence, risk, social, peer

## Abstract

Adolescence is a period of escalated rates of risk taking and a dynamic social landscape with peers taking on an important role in shaping one’s decisions. Choosing to engage in risk rarely impacts only the decision maker, but also those around them. With a cohort of typically developing adolescent and young adult friend dyads (*N* = 128, 11–22 years), the current study investigates how peer-relevant social contexts influence risk preferences at different ages using a computational decision making task. We adapted a computational expected utility model to account for weighing the friend’s outcome as part of one’s utility calculation when deciding between assigning the risky option to oneself or one’s friend. Compared to participants’ baseline risk preferences absent of any friend involvement, we found age-related changes in risk taking when the preferred option can only be assigned to oneself or one’s friend but not to both. Exploratory, data-driven analyses using behavioral measures and the computationally derived risk preference parameter revealed that overall, early adolescence is a period in which individuals assigned more weight to their friends’ outcomes and were willing to forego personal benefits to a greater extent. Active observation by friends had no additional, age-dependent impact on participants’ risky choices. These results indicate early adolescence to be a period of sensitivity to social contexts evoking prosocial gestures that are costly to oneself.

## INTRODUCTION

Adolescence, the phase of life between childhood and adulthood that starts with physical puberty (e.g., Spear, 2000), has long been a focus in public health and policy due to it being a period of escalated rates of participation in health risk behaviors (Boyer, 2006; Eaton et al., 2010; Steinberg, 2008) and self-reported risk taking propensity (Tervo-Clemmens et al., 2024). A meta-analysis of laboratory based risky decision tasks showed that adolescents take more risks than adults (Defoe et al., 2015), in line with public health data. In continuing to understand the mechanisms underlying heightened risk taking during adolescence, it is beneficial to zoom out and appreciate its multifaceted nature. In adolescence, people begin to spend more time with their peers and less time with their parents (Barnes et al., 2007), thereby shifting their social groups from family- to peer-oriented, with less parental supervision (Rubin et al., 2008; Steinberg & Morris, 2001). This evolving social environment allows them more agency and thus opportunities to take risks that impact their and their peers’ wellbeing, which may in turn influence their relationships (Blakemore, 2018; Blakemore & Mills, 2014).

The present study examines possible roles that peers play in shaping risk-taking during adolescence. Adolescents increasingly value peer acceptance (Brown, 2004; Nelson et al., 2005; Guyer et al., 2012; Rodman et al., 2017; Silk et al., 2012; Somerville et al., 2013), and start to adopt more other-focused, prosocial behaviors (Crone & Dahl, 2012). The social consequences of one’s risky acts for one’s peers could be a potent factor in shaping adolescents’ behavior (Powers et al., 2018), and several motives may underlie peer-related shifts in decision making. For example, they are prone to behave in a riskier manner around peers they perceive, correctly or incorrectly, as more risk accepting (Knoll et al., 2015; Prentice & Miller, 1993). Peer involvement may also give rise to increased risk taking because it might amplify the neural responses signaling potential rewards (Albert et al., 2013; Chein et al., 2011), though the evidence is mixed (Hinnant et al., 2019). Prosocial motivations to help others could also underlie increase risk taking in adolescents in social contexts in which risk leads to beneficial outcomes for others (Do et al., 2017). Moreover, the expression of these motives may vary across adolescence due to these evolving social dynamics (e.g., Lam et al., 2014), and/or the still-changing neurocognitive processes influencing decision making more generally (Hartley & Somerville, 2015).

In an effort to delineate how different social contexts shape adolescents’ risk preferences, the present study induces differing social consequences of risky behavior between pairs of friends and uses computational analyses to isolate whether and how they shape risk preferences across adolescence to young adulthood. Risk taking hereafter is defined following the Judgement and Decision Making literature, as taking an action for which the outcome is variable, with greater variability indicating more risk (Weber et al., 2004), as opposed to the lay conception that risk refers to harmful behaviors that ought to be minimized.

The present study uses two “friend outcome” conditions that manipulate whether engaging in a risky act is differentially advantageous to oneself relative to one’s friend. One condition (*Identical*) involves participants making a risky decision that will subject their friend to the same outcome as themselves to mimic situations in which individuals are engaged in risks with joint outcomes (e.g., making a risky but potentially rewarding move in a team sport, signing your student band up for a contest). The other condition (*Opposite*) sets up opposing outcomes for the dyad. There is a risky and a safe option – one of which is preferred to the participant – and the participant must decide whether to assign that option to themself or their friend. This mimics situations in which taking a risk could disadvantage a friend (e.g., running for student body president when your friend is also running). Prior work has shown that when a friend stood to benefit from a participant’s choice, adolescents shifted their risk preferences regardless of the friend witnessing the decision or not, while adults did so to a greater extent while being observed (Powers et al., 2018). The current study advances this prior work by using two distinct social-motivational contexts to examine whether individuals’ prosocial behavior persists when social consequences of their risky decisions vary in how the participant’s choice impacts their friend’s outcome.

In the present study, the social outcomes described above were embedded within a second social context manipulation – whether those decisions were made while the friend actively observed the participant’s choices. Prior work has examined whether adolescents take more risks when peers were present and observing an individual’s choices, as is observed in real-world scenarios like driving (Eaton et al., 2010; Gardner & Steinberg, 2005). While several studies have found an increase in risky choice behavior under observation (Gardner & Steinberg, 2005; Smith et al., 2015; Van Hoorn et al., 2017), a recent meta-analysis has found that the basic effect of observation was rather small and context-dependent (Powers et al., 2022). The present study uses friend observation to examine the extent to which participants are more likely to maximize their friend’s potential gains when they are actively observing the choice. Incorporating this condition is poised to clarify the underlying motives of the decision maker. If an individual chooses to give a peer an advantageous option more when they are watching, it implies a reputational motive is underlying the prosocial act.

Thus, the current study aims to investigate age-related changes from adolescence to early adulthood in how peer outcome in different social contexts influences individuals’ risk preferences, compared to when the decision only impacts oneself. Peers in this study were age- and gender-matched friends rather than strangers to evoke the possibility of genuine relational motives. To measure risky decisions, we used an economic decision making task in which participants first chose between risky (the option with more variable outcomes) and safe options absent friend involvement, allowing us to compute their baseline risk preferences. We then introduced the *Identical* and *Opposite peer outcome* conditions described above. A computational model was used to derive estimates of risk preferences at baseline (absent friend involvement), and in each of the peer outcome conditions, further stratified by whether the friend was observing the choices. By evaluating age-related changes in risk preferences in these contexts under *observation* or *no observation* by the friend, we can learn what kinds of social considerations and potential consequences might motivate individuals to shift their decisions across adolescence.

## METHODS

An extended version of this project is preregistered at https://osf.io/9brsg/.

### Participants

Participants (age range: 12.0–22.8 years) were recruited through an existing database, advertisements, and community outreach. Participants were required to be based in the United States, fluent in English, and have access to computer set-up to complete the study. Individuals meeting these inclusion criteria were then asked by researchers to invite a friend of the same sex and within a year of the same age to contact the lab to indicate their interest in co-participating. If their friend was also eligible and interested in the study, the pair was tested. During the screening process we excluded potential participants who had learning disabilities, and who had previously participated in a peer-related study in the lab. Within each friend pair, we collected fully mutual data in all conditions – each individual took on the role of the primary decision maker and the role of friend (for the other person in the dyad).

Overall, 128 participants took part in this study in pairs (*N* = 37 adolescent pairs, ages 11.98–17.46 years; 18 pairs female and 19 pairs male; *N* = 27 young adult pairs; ages 18.16–22.82 years, 14 pairs female, 13 pairs male; 15.6% Asian, 6.3% Black or African American, 10.2% two or more races, 0.8% other, 0.8% Native Hawaiian or Pacific Islander, 66.4% White. One participant from the adolescent group identified as gender non-conforming and was the peer partner of a male-identifying participant, and one participant from the adult group identified as transgender male and was the peer partner of a male participant.

Male and female pairs were distributed evenly across the age range (logistic regression with age predicting gender, *B* = −0.014, *p* = .789*)* and pairs had an average age difference of 0.46 years (min = 0.01, max = 1.39, *SD* = 0.33). The distribution of average parental education level as a proxy for socioeconomic status (SES) was not related to age (linear regression, *B* = −0.016, *p* = .439), indicating SES is balanced across age. Participants provided informed written consent/assent, and parents/caregivers of minors gave written permission for their participation. The Committee for the Protection of Human Subjects at Harvard University approved this research.

### Sample Size and Exclusions

Sample size was determined prior to data collection based effect sizes from Powers et al. (2018): The effect size (*r* = .32) for the significant comparison between adults and adolescents in the influence of age on risk aversion (Alpha) when facilitating positive outcomes for peers, and effect sizes ranged between *r* = .25–.32 for age effects within the adolescent subgroup (continuous age-related changes). Using the lowest effect size *r* = .25, a power analysis using the package *pwr* in R (v1.3.0, Champely et al., 2020) showed that *N* = 123 participants would be needed for 80% power to detect this effect size in the current study. Our study deviates from Powers et al. (2018) in analytical framework. Due to the nonparametric nature of spline terms from the GAM models, we could not perform a power analysis reflecting the nonparametric analyses used without making strong (parametric) assumptions about the functional form of the smooth. Powers et al. (2018) was selected for its conceptual relevance, and therefore, discussion of power should be considered more illustrative than prescriptive.

Part 1 of the study included usable data from *N* = 128 participants. Part 2 of the study included usable data from *N* = 123 participants (3 participants failed the comprehension checks and did not call the experimenter, and 1 additional pair that had technical issues resulting in data loss, were excluded).

### Risky Decision Making Task

Participants completed an economic decision making task consisting of different choice options varying in monetary payout and odds of winning, which had been used in previous studies to quantify individuals’ risk preferences in adult (Chung et al., 2015; Levy et al., 2010; Powers et al., 2018; Sip et al., 2015; Tymula et al., 2012) and adolescent samples (Powers et al., 2018; Tymula et al., 2012). We adopt the definition of risk taking from the decision making literature (e.g., Weber et al., 2004) based on outcome variability (i.e., riskier options are those with more variance in potential outcomes), an approach that is agnostic with respect to whether risks are good or bad.

**Figure 1.**
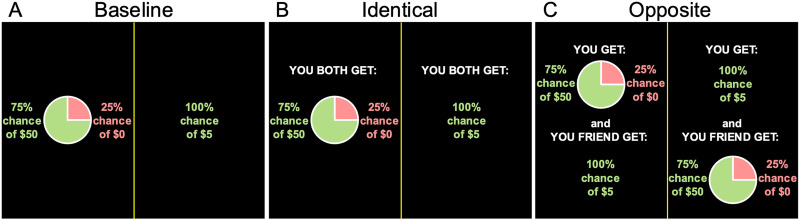
Example trials from different conditions in the experimental task. A) Baseline: a choice between a safe option (100% chance of winning $5) and a risky option (75% chance of winning $50, 25% chance of winning $0). B) Identical: participant and friend receive the same outcome chosen between the two options described. C) Opposite: one person receives the safe or risky option, and the other will receive the unchosen option.

On each trial, participants selected between two choice options displayed on the left and right sides of the screen (Figure 1) varying in risk: a) a safe option “100% chance of winning $5” and b) a risky lottery option (10%, 25%, 50%, 75%, or 90% probability of winning [$5, $10, $20, $30, $40, $50, $100]; Powers et al., 2018; Tymula et al., 2012) depicted with a pie chart indicating the odds of winning and losing. Each probability was paired with each amount totaling 35 trials per condition, with the order of trials randomized. The safe and risky options were counterbalanced to appear on the left and right sides of the screen at equal frequency. For all conditions, participants indicated their choice in self-paced timing by pressing one of two keyboard buttons, at which point the trial ended; there was no immediate feedback on the outcome of the risky choice.

Participants were instructed that they and their friend would receive a fixed percentage of the cumulative amount of money they earned through their choices as a bonus, with a computer simulating the chosen lotteries to determine earnings. Participants in the “friend” role were paid the percentage from the Identical and Opposite condition based on choices made for them by the participant in the primary decision maker role.

### Experimental Procedure

Following screening and consenting, each participant first completed an online testing session from their individual computer (“Part 1”) on Qualtrics in which they completed several iterations of the risky decision making task individually (described below). Once both participants in a dyad had completed their individual session, the friend dyads co-participated in the second part of the study (“Part 2”) on Zoom with the experimenter, where each friend logged in separately. Screen sharing and camera status was manipulated to control whether the decision maker’s friend was observing them in real time (Observed vs. Unobserved).

#### Part 1: Individual Participation.

Each participant in the pair completed Part 1 separately and not necessarily on the same day. Participants received detailed instructions and underwent comprehension checks which assessed their comprehension of the probability graphics used in the study before they could start the self-paced task. They completed the following conditions in pseudorandomized order. In each condition, participants viewed the same risky options described above under the following conditions:

1. Baseline: Participants completed risky decision making task described above with outcomes for themselves only (Figure 1A). The purpose of this condition was to establish participants’ individual risk preferences. We also collected data on what participants predicted their friend would choose in this condition, which was only used here as part of the input for the computational model for the Opposite condition (detailed in model-based estimation of risk preferences section below).
2. Identical: Participants completed the risky decision making task described above knowing that their selection would apply to both themselves and their friend (Figure 1B). The purpose of this condition was to show how participants’ risk preferences changed relative to Baseline, when their decisions would subject their friends to experiencing the same outcome.
3. Opposite: Participants completed the risky decision making task described above and selected an outcome for themselves with the knowledge that the unselected option would apply to their friend (Figure 1C). The purpose of this condition was to show how participants’ risk preferences changed relative to Baseline, when participants either had to keep or give their preferred option to their friend.

#### Part 2: Dyad Co-participation.

The second part of the study was conducted with each member of a dyad plus the researcher meeting on Zoom. Friend observation was manipulated as follows: For the Observed conditions, both the participant and their friend had their cameras on, and the participant shared their screen so that their friend could observe them making decisions in real time. For Unobserved conditions, the screen was not shared, and the friend would turn off their camera.

To examine the impact of friend observation in both the identical and the opposite contexts, this part of the study included four conditions: Observed Identical, Unobserved Identical, Observed Opposite, Unobserved Opposite. These conditions were administered in interspersed blocks with randomized order.

### Dependent Measures

1. Model-based estimation of risk preferences: we fit a probabilistic choice model with two free parameters to each participant’s choices for each condition to quantify individual risk preferences (Gilaie-Dotan et al., 2014; Levy & Glimcher, 2011; Levy et al., 2010, 2012). As in Powers et al. (2018), we modeled the expected utility of each option with a power utility function:

EU=pvαoriginal model

where *v* is the amount of money on a given choice and *p* the probability associated with winning that dollar amount. Alpha is the estimated parameter of interest for risk preference, where risk is defined as outcome variability (Weber et al., 2004), i.e., the possible outcome with greater uncertainty is the riskier choice. Alpha < 1 indicates an agent is risk averse with a concave utility function, preferring the safe option to a risky option with equal expected value (EV; dollar amount multiplied by the probability of winning that option). Overall, prior work has shown that most people are risk averse on average (Glimcher, 2008; Platt & Huettel, 2008). A score of Alpha > 1 indicates an agent is risk seeking with a convex utility function, preferring the risky option to the safe option with equal EV. Alpha = 1 indicates a risk neutral agent with a linear utility function, who is agnostic between the safe and risky options with the same expected value. In Supplementary Material, we compared the Expected Utility model with the alternative Prospect Theory (Tversky & Kahneman, 1992) models, and demonstrated that, consistent with previous studies (Levy & Glimcher, 2011; Levy et al., 2012; Powers et al., 2018; Tymula et al., 2012), the Expected Utility model is favorable and therefore used for all reported analyses.

These valuation processes require three inputs each (probability, value, and Alpha). Additionally, the Opposite condition required an adaptation of the model to integrate the utility of the chosen option (the one participants assigned for themselves) relative to the utility of the unchosen option (the one participants assigned to their friend). Thus, we adapted the expected utility model to reflect this adjudication.

The probability and value inputs for the friend were derived from the options themselves. For the Alpha parameter computing the utility of the option assigned to their friend, we estimated the participant’s conception of their friend’s risk preference using additional data collected in Part 1 in which participants predicted their friend’s trial-by-trial choices. These data were acquired similarly to the Baseline condition during the study’s Part 1 but instead participants answered the question “What do you think your Friend would choose?”. This permitted calculating what the participant believed was their friend’s Baseline risk preference (Alpha*_friend_*). In addition, a new parameter *w* was added as follows, rendering the expected utility of a chosen option as a weighted average for oneself and one’s friend:

EU=1−wpselfvselfα+wpfriendvfriendαfriendrevised model

where *p*_*self*_ and *v*_*self*_ are the probability and amount of money associated with the option participants intend to choose for themselves and *p*_*friend*_ and *v*_*friend*_ are associated with the unchosen option which would be assigned to their friend. Alpha is still the free parameter to be estimated for participants’ risk preferences, and Alpha*_friend_* is participants' estimated risk preference for their friend, which is an individualized constant derived from fitting the original model on each participant’s predicted friend choices in the Baseline condition (see Supplemental Materials for report on age trends in Alpha*_friend_*). Weight_friend_ (*w)* represents how much one’s utility is based on one’s own outcome relative to how much one incorporates their estimation of their friend’s outcome and is bounded between 0 and 1. The larger it is, the more the participant values the utility of their friend’s outcome relative to their own.

With both the original and revised model, we fit each trial within a condition from a given participant to a logistic function that computes the probability of the participant choosing the risky option on that trial given the expected utility of the safe and the risky option, with a maximum likelihood procedure:

$$P\left(\text{choose risky option}\right)=\frac{1}{1+\exp(\beta ({EU}_{\text{safe}}-{EU}_{\text{risky}}))}\;$$

where *EU*_*safe*_ is the expected utility of the safe option and *EU*_*risky*_ is the expected utility of the risky option. Beta is the inverse decision noise, which indicates the likelihood of the participants choosing the option with the higher expected utility as a theoretical rational agent would. Higher Beta estimates indicate the participant's choices are more yoked to expected utility, whereas lower Beta indicates more stochastic choices that are less yoked to expected utility.

Parameter recovery showed that both models recovered robustly. Model comparison showed that both outperformed alternative models for their respective conditions. See Supplemental Materials for more detail on these two procedures.

The computational models were fitted to each participant’s data individually rather than hierarchically to avoid any a priori age assumptions having undue influence on the subsequent age-based analyses. Defining age groups during this step is incompatible with our goal of avoiding the need to arbitrarily group participants based on age bands. This is consistent with prior developmental work that also did not adopt a hierarchical framework (e.g., Master et al., 2020; Nussenbaum et al., 2022; Powers et al., 2018). Fitting the computational model to participant data individually without pre-imposed age boundaries allows the generalized additive models to capture age-related change in a continuous and nonlinear manner in our statistical inferences.

#### Model-Based Exclusions.

For the analyses described above that use the Alpha, for Baseline and any condition involving the Identical context, we excluded participants with Alpha exceeding 3.32, which is the maximum theoretical recoverable value based on our choice set; for conditions involving the Opposite contexts, we excluded participants whose Alpha exceeded 2 or Weight_friend_ lower than 0.000001. This threshold is set based on two considerations. First, the choice behavior generated by Alpha above 2 were barely distinguishable from each other, with the maximum difference in one choice (see Supplemental Materials for more details). Second, the revised model showed the best parameter recovery for Alpha in the range of 0 to 2 (correlation between simulated and fitted alpha in the range of [0,2]: r = 0.78, Weight_friend_: r = 0.73). We also excluded participants whose AIC from the computational model was equal to or exceeded the AIC value for someone with chance behavior from that condition. Model based exclusion resulted in exclusion rate of less than 10% (see Supplemental Materials).

For completeness, we included results using a non-model based alternative measure to Alpha, proportion of risky choices (calculated as the number of risky options chosen divided by total number of trials within a condition for each participant), in Supplemental Materials. This measure does not take into account the relative utility of the options on each trial and is thus a more coarse-grained measure than Alpha. However, it is not subject to model-based exclusions and provides a sketch of the raw data. We also report a set of sensitivity analyses in Supplemental Materials, demonstrating that these exclusions did not alter the inferences substantively from using the full sample.

2. Measure of simulated earnings: Simulated earnings were computed for each participant for each condition by calculating the sum of the expected value (EV) of each selected choice option. Simulated earnings were modeled as proportions of the total possible earnings (calculated as the sum of all highest-EV choices) using a Beta distribution to better reflect the distribution of the data and improve the accuracy of the model fit and generalizability. Therefore, the test statistic is expressed in *z*-values and the resulting plots with *y*-axis on a logit scale.

### Analysis

The research questions under investigation aim to capture the risk preferences in adolescents and adults and the degree to which different social-motivational contexts influenced their risky decision making. The research questions and analytical plans for each are described below following a description of how all analyses treat age. For all group analyses, the random effect structure treated participant as nested within a dyad (each representing a friend pair) to account for any dependencies related to the data being collected on pairs of friends.

#### Treatment of Age.

Because risk preferences have been found to follow a nonlinear pattern from childhood to adulthood (Casey et al., 2011; Cauffman et al., 2010; Figner et al., 2009; Galvan et al., 2007), we adopted an exploratory data driven approach sensitive to nonlinear age related changes. Specifically, we fit a generalized additive model (GAM) (Hastie & Tibshirani, 1986, 1990) using the *gam* function from the *mgcv* package (v1.8.38, Wood, 2003, 2004, 2011, 2017) to examine the effect of age as a continuous predictor on the dependent variables (DVs).

In order to identify age windows where the GAM model shows significant changes in the DV with respect to age as the only predictor, we computed the first derivatives of the GAM curve (spline) along the *x*-axis (age), which quantified the slope of the curve at each given age, using the *gratia* package in R (v0.7.3.7, Simpson, 2022). The derivatives were evaluated along 100 points along the *x*-axis and Simultaneous Confidence Intervals (CIs) corrected for multiple comparisons. Quantifying the age windows at which the Simultaneous CIs of the first derivative did not include 0 reveals the slope across age was significant. Positive values indicate a positive slope or an increase in the DV, negative values indicate a negative slope or a decrease in the DV, and 0 indicates the DV remains constant.

To compare the age pattern for different levels of a categorical variable, GAM fits a nonlinear function for each level of a categorical variable (factor smooth interaction) without providing a direct statistical test for the interaction between the smoothed variable and the categorical predictor of interest. Therefore, we used the *plot_diff* function from the *itsadug* package (v 2.4, van Rij et al., 2022) to inspect the relative difference between fit estimates of each pair of conditions (i.e., Identical vs. Baseline, Opposite vs. Baseline) and compute 95% simultaneous CIs around it. Age bands where the simultaneous CI did not contain zero are interpreted as periods of significant differences between the conditions.

We applied the analytical methods above to evaluate our central research questions:

1. *How do risk preferences change across age from adolescence to young adulthood?* We fit a GAM and examined the first derivatives as described above to test the effect of age as a continuous predictor and characterize age-related changes in choices made in the Baseline condition.
2. *Does offering identical or opposing outcomes for oneself and one’s friend change one’s risk preferences, compared to when the outcome only concerned oneself? Are there age-related differences in this effect?* We fit a GAM and used factor smooth interactions as described above to examine the effect of age as a continuous predictor and characterize age-related changes in choices made in the Identical and Opposite conditions compared to Baseline. We did not conduct direct comparisons between the Opposite and the Identical context because the two conditions are not matched on low-level features (the Opposite context had more information to consider and thus likely required greater cognitive demand to simulate the two different scenarios for self and friend).
3. *Does being observed by friends change participants’ risk preferences and simulated earnings in Identical and Opposite contexts? Are there age-related differences for this effect?* We fit a GAM using age as a continuous predictor and a 4-level factor predictor “Condition” (Observed-Identical, Unobserved-Identical, Observed-Opposite, Unobserved-Opposite) and used factor smooth interactions described above to evaluate age-related changes due to observation within the Identical and Opposite contexts, respectively. This was done instead of testing the interaction of Identical and Opposite conditions and observation, due to the aforementioned difference in cognitive demand between the Identical and Opposite context.
4. *Are there age-related changes in Weight*_*friend*_ *(how much the friend’s outcome was taken into account in one’s decision)?* We used the same analytical approach outlined in 1) to investigate effects of age on how much individuals factor their friend’s utility into their decisions. This analysis is naturally constrained to the conditions fit with the revised model, i.e., the Opposite context conditions in which each member of the dyad receives different outcomes, as the *Weight*_*friend*_ is not part of the original model. The original model fit best for the Baseline and Identical context conditions, and the revised model fit best for the Opposite context conditions (see Supplemental Materials).

## RESULTS

### Baseline Risky Decisions

This set of analyses evaluated the relationship between risk seeking and age, and between simulated earnings and age, in the Baseline condition. The sample was risk averse on average (Mean Alpha = 0.55, *SD* = 0.23), in line with prior results in different populations including adolescents (Blankenstein et al., 2016; Gilaie-Dotan et al., 2014; Kahneman & Tversky, 1979; I. Levy et al., 2010; Pratt & Zeckhauser, 1987; Tymula et al., 2012). Simulated earnings indicated participants incorporated expected value into their choices (Mean simulated earnings: $614.52 out of $662.50 maximum earnings, *SD* = 63.74). The simultaneous CI of the first derivative of the spline included 0 at all ages for both Alpha and simulated earnings. This indicates that participants’ risk preferences and the extent to which they maximized potential payouts remained consistent across the age range of 12.0–22.8 years.

### Age-Related Shifts in Risky Choice Invoked by Identical Friend Outcome

Examining the Identical condition tested whether joint outcomes (risky choice having the same impact on oneself and one’s friend) influences risk preferences relative to one’s baseline. There were no differences in Alpha between the Identical condition and Baseline overall, or age-related changes (Mean of Identical vs. Baseline: 0.59 vs. 0.55; *B* = 0.04, *t* = 1.61, *SE* = 0.03, *p* = .108). The same was true when examining the proportion of simulated earnings (Mean of Identical vs. Baseline: .93 vs. .92; *B* = 0.08, *z* = 0.76, *SE* = 0.11, *p* = .448). This indicates that individuals judge risk similarly when it stands to benefit oneself only, and oneself and one’s friend.

### Age-Related Shifts in Risky Choice Invoked by Opposite Friend Outcome

#### 1) Risk Seeking.

**Figure 2.**
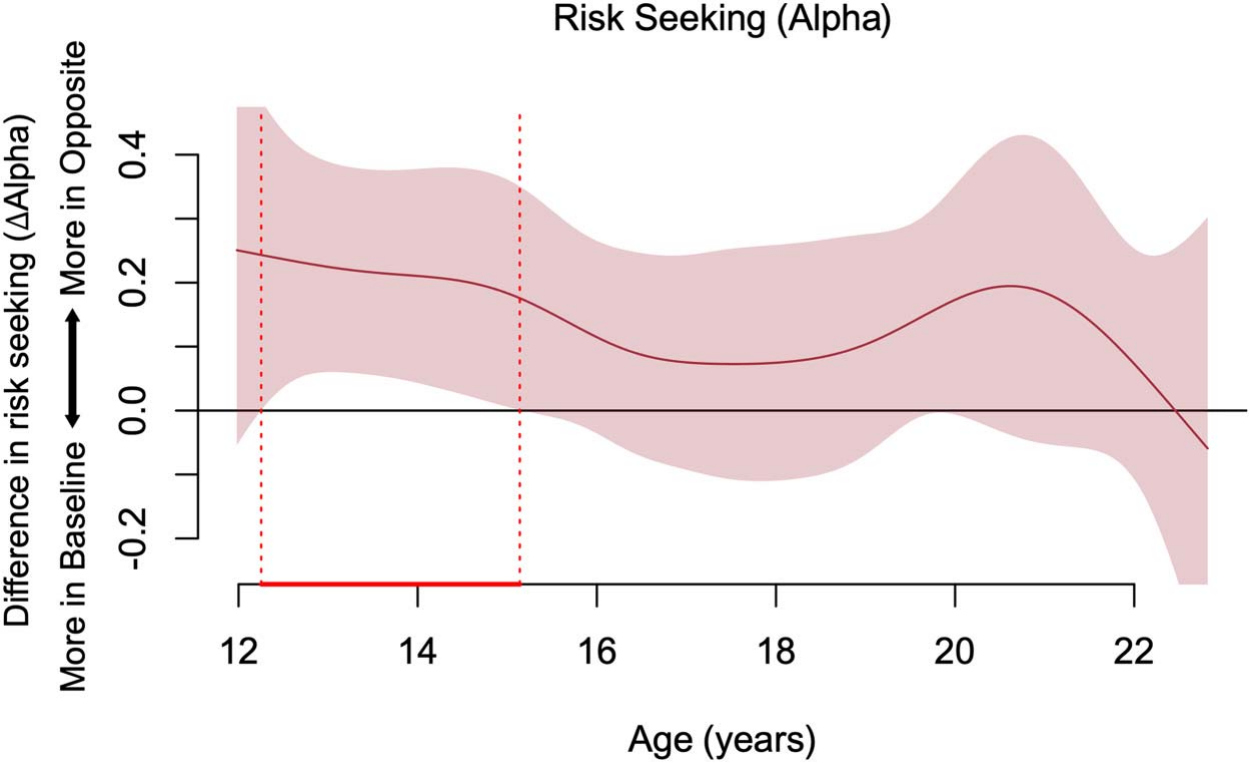
Age-related patterns of risk seeking: difference of fit estimates of the GAM model. Positive values indicate risk seeking is greater at Opposite than Baseline, negative values indicate the reverse, and 0 indicates risk seeking is not significantly different across the conditions. Shaded area represents 95% simultaneous CIs of the difference between fit estimates at each age point. Dotted red lines show that between ages 12.3–15.1 years, the 95% simultaneous CIs do not include 0, indicating significant age-related changes.

In the Opposite condition, participants’ choices caused their friends to receive their unchosen option. Analyses determined the extent to which participants changed their Baseline risk preferences when their friend stood to reap the opposite of what they chose for themselves. Overall, participants were relatively more risk accepting for themselves when it meant that their friend would otherwise have to accept the risky option (Mean of Opposite vs. Baseline: 0.69 vs. 0.55; *B* = 0.14, *t* = 5.25, *SE* = 0.03, *p* < .001). There was a significant age-related difference in this tendency, such that the overall effect was evident during ages 12.3 to 15.1 years (Figure 2). Participants’ risk preferences were not significantly different for Opposite and Baseline conditions in the rest of the age range. Thus, from early to mid-adolescence, individuals were relatively more risk accepting when they had to decide whether to keep their preferred option or assign it to their friend instead.

#### 2) Simulated Earnings.

To examine the effect of the Opposite condition vs. Baseline on simulated earnings, we used the same analytical approach as above. Compared to Baseline, participants earned less in the Opposite condition, when their decisions caused their friends to receive the opposite of their own choices (Mean of Opposite vs. Baseline: .79 vs. .92; *B* = −0.81, *z* = −7.89, *SE* = 0.10, *p* < .001).

**Figure 3.**
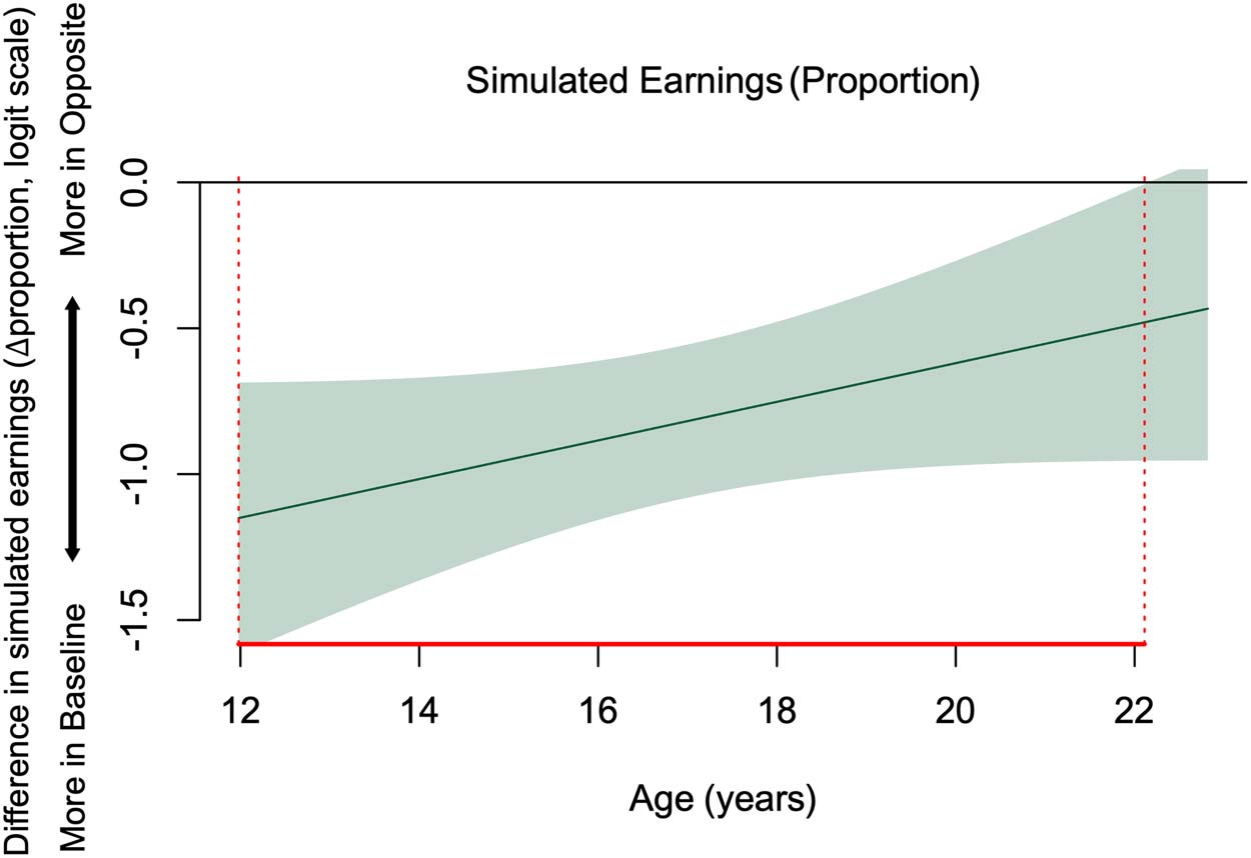
Age-related patterns of proportion of simulated earnings: difference of fit estimates of the GAM model. Negative values indicate the amount earned was less for Opposite than Baseline, positive values indicate the reverse, and 0 indicates the simulated earnings are not significantly different across the conditions. Shaded area represents 95% simultaneous CIs. Dotted red lines show that between ages 12.0 and 22.1, the 95% simultaneous CIs do not include 0, indicating significant age-related changes.

There was a significant age-related difference in the proportion of simulated earnings between age 12.0 and 22.1 years (Figure 3). Participants from this age range earned less in the Opposite condition than in the Baseline condition and this difference diminished with increasing age. Thus, from early adolescence to young adulthood, individuals incurred personal loss when they faced the choice of keeping their preferred options or assigning those to their friend instead.

#### 3) How Much Friend Outcome Weighs Into Choices (Weight_friend_) in the Opposite Condition.

**Figure 4.**
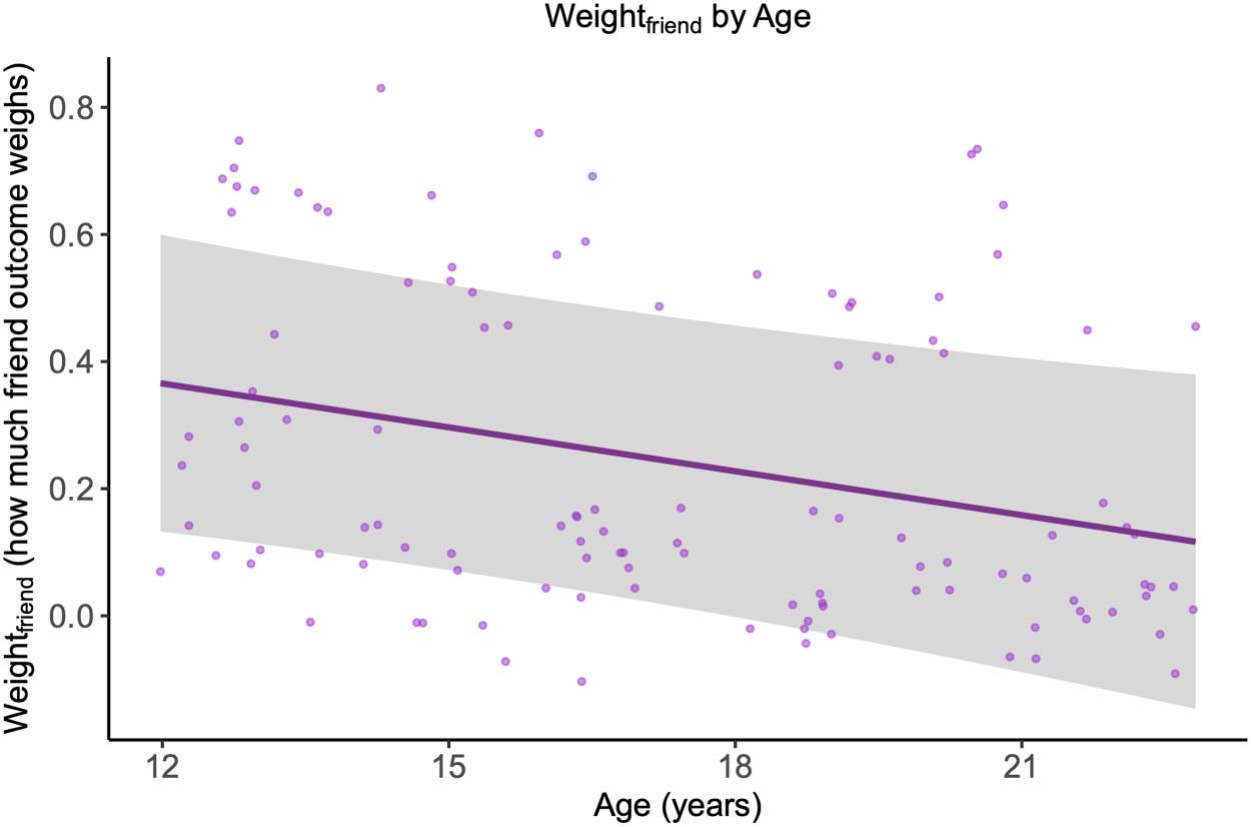
Age-related changes in Weight_friend_. The purple line with shaded area representing 95% CI represents the model expected value for the weight assigned to the friend's outcome across the age range. Dots represent individual data points.

In the Opposite condition, there was a linear decline in how much the friend’s outcome weighed in individuals’ decisions (*edf* = 1, *F* = 5.89, *p* = .017, Figure 4). The younger the participant, the more they factored their friend’s outcome into their risky decision and consequently downweighed their own.

### Age-Related Shifts in Risky Choice Evoked by Friend Observation

#### 1) Risk Seeking.

Based on estimates of Alpha, participants were relatively more risk seeking in the Observed Identical condition, when friends watched them make decisions that yielded the same outcomes for them both, than in the Unobserved Identical condition, when the participants made the same type of decision when not being observed (Mean of Observed vs. Unobserved: 1.04 vs. 0.94; *B* = 0.10, *t* = 2.65, *SE* = 0.04, *p* = .008). In the Opposite context, there were no significant differences when directly comparing Observed vs. Unobserved conditions (Mean of Observed vs. Unobserved: 0.69 vs. 0.69; *B* = −0.001, *t* = −0.04, *SE* = 0.04, *p* = .971). No age-related differences were found for the effects of observation in either Identical or Opposite contexts.

#### 2) Simulated Earnings.

There were no main effects or age-related differences on the proportion of simulated earnings as a function of observation in either the Identical context (Mean of Observed vs. Unobserved: .95 vs. .95; *B* = −0.003, *z* = −0.02, *SE* = 0.10, *p* = .981) or the Opposite context (Mean of Observed vs. Unobserved: .75 vs. .77; *B* = −0.09, *z* = −1.06, *SE* = 0.08, *p* = .289). Having participants’ friends observe their choices led to an overall slight rise in risk seeking, but this overall effect was consistent across conditions and ages.

#### 3) Friend Outcome Weight (Weight_friend_) in the Observed Opposite Condition.

There were no main effects of observation on age-related changes in how much friends’ utility was factored into one’s decisions (Mean of Observed vs. Unobserved: 0.34 vs. 0.33; *B* = 0.02, *t* = 1.16, *SE* = 0.02, *p* = .249).

## DISCUSSION

The goal of this study was to examine how social contexts shape risky decisions in individuals varying in age from early adolescence to young adulthood. Using an economic decision task and dyads of friends making decisions in social contexts where their choices differentially impacted themselves and their friends, with a subset of the contexts involving friend observation, we delineate how these factors differentially impact participants’ risky choices. We found that these social contexts variably motivated participants to adjust their decisions relative to their own baseline risk preferences. Analyses of continuous age that were sensitive to nonlinear changes isolated the age windows of sensitivity in which their risk preferences were modulated by the social contexts. Overall, we observed early adolescence to be an age of particular susceptibility to these forms of social influence: individuals were more willing to take disadvantageous risks to protect their friends, which cost them earnings. This effect was further corroborated in computational modeling analyses showing that more weight was placed on their friend’s outcome in younger participants. This suggests that early adolescents are willing to yield their baseline preferences to offer an advantage to their friend, even if it means incurring more risk than they are naturally inclined to take when the outcome only impacted themselves.

### Baseline Risk Preferences

In the Baseline condition, the outcome of risky choice solely concerned oneself. As with several previous risky decision making tasks (Braams et al., 2019; I. Levy et al., 2010; Powers et al., 2018; Tymula et al., 2012), in ours, taking risks (the option with variable outcomes) was on average more economically advantageous than not doing so. This design was intended to encourage participants to make risky choices, since people are overall risk averse (Blankenstein et al., 2016; Gilaie-Dotan et al., 2014; Kahneman & Tversky, 1979; I. Levy et al., 2010; Pratt & Zeckhauser, 1987; Tymula et al., 2012), which is the case in our sample as well. We did not observe any age-related changes in risk aversion or the extent to which participants maximized earnings. This lack of age-related changes in risk taking is in line with some prior work showing that when by themselves, adolescents and adults do not differ in their levels of risk taking (Blankenstein et al., 2016; Gardner & Steinberg, 2005; Van Leijenhorst et al., 2008).

That said, there is heterogeneity in age patterns in risk taking across laboratory tasks, despite an overall trend toward adolescents taking more risks than adults (Defoe et al., 2015). For example, Blankenstein et al. (2016) and Tymula et al. (2012) showed adolescents preferred fewer risky options than adults. Potential factors contributing to not observing an adolescent peak in risk preferences include the probabilities used in this study being known rather than ambiguous, and the absence of immediate feedback on the outcome of the choices, both of which have been suggested as drivers of heightened risk taking in adolescence (Blankenstein et al., 2016; Defoe et al., 2019; Tymula et al., 2012). Description-based tasks like the current task (where the probability and outcome information is available to participants) are less likely to yield an elevation in risk taking in adolescence compared to experience-based tasks where such information is learned through trial-and-error (Rosenbaum et al., 2018). Future, well-powered studies are needed to clarify which age-related changes in risk preferences are robust.

### Influence of Friend Outcome

In the Opposite context, participants faced the dilemma to keep or assign their preferred option to their friend. This manipulation elicited a robust shift in risky choices. Participants overall were relatively more risk accepting but earned less in this context compared to Baseline when decisions only concerned themselves. This implies their objective was not solely maximizing earnings and that social considerations were at play. Age-related changes in risk preferences were observed at ages 12.3–15.1 years, where participants were relatively more risk accepting and willing to take more economically disadvantageous risks, presumably to improve their friend’s outcome.

The fact that younger adolescents prioritize peers’ outcomes is a testament to the potency of peer relationships at this age. The earlier age window aligns with past work showing that early adolescents are more sensitive to social feedback (Rodman et al., 2017), the perspectives of others in prosocial decision making (van den Bos et al., 2011), and that anticipation of social-evaluative contexts can induce heightened arousal and self-conscious emotions (Somerville, 2013). Thus, participants in this age range might be more motivated to shift their behavior in a direction that benefited their friend due to newly heightened concern for positive standing with peers.

Further, emerging research suggests that cognitive and neurodevelopment through adolescence ushers in more sophisticated strategic cognition that allows for calibration of behavior to multiple, sometimes competing, information streams to maximize goal-directed actions (Hartley & Somerville, 2015; Jacobs & Klaczynski, 2002; Wilbrecht & Davidow, 2024). Applied to this particular study, we speculate that an increasing capacity for this form of strategic cognition could help a decision maker optimize self- and friend-accommodating motives simultaneously. The older adolescent participants’ choices also shifted toward accommodating their peers, but more selectively and while losing fewer earnings. Young adolescents’ tendency to downweigh self-focused outcomes in favor of peer-focused outcomes could therefore reflect an ongoing fine-tuning of strategic choice behavior.

We modified the computational model of expected utility to mirror the adjudication between assigning a risky option to oneself or one’s friend. A new parameter, Weight_friend_, was introduced to reflect how much participants weighed their friend’s possible outcome into their choice about who will receive the risky option. The original model that did not incorporate the friend’s outcome was not able to reliably capture participants’ behavior in the Opposite context (see Supplemental Materials). Thus, treating the friend’s outcome as if it is part of one’s own utility calculation is an important factor for modeling a decision process that involved opposing interests between friend and self. The data thus suggest that participants were optimizing for two sources of utility: financial and social, and that they took their friend’s preference into account in their decision making process. This is in line with past work demonstrating that social-motivational contexts ought to be taken into account in understanding heterogenous (across decision contexts and individuals) patterns in altruistic (sacrificing recourses to benefit others) behaviors (Andreoni & Miller, 2002).

The revised computational model provided additional insight in age-related changes in how much people weighed their friends’ outcome in the Opposite condition. Weighing friends’ outcomes declined linearly with increasing age. This finding dovetails with the finding that participants earned less in this condition, and this difference diminished with increasing age. Together, these findings show that younger participants placed more weight on their friends’ outcomes and were willing to forego personal benefits to a greater extent than older participants.

Studies have shown that strategic prosocial behaviors (in which being prosocial is at least partly motivated by maximizing one’s own gains) increase with age during adolescence through development in perspective taking (Crone & Achterberg, 2022; Güroğlu et al., 2014; Overgaauw et al., 2012; Steinbeis et al., 2012). In the Opposite condition, with increasing age, participants lost less money compared to the Baseline condition, suggesting older participants were more strategic and mindful of the payout when changing their preferences. This result also echoes the finding from Powers et al. (2018) that participants incurred losses to protect their friend when their friend could potentially suffer loss due to their decision. Our findings suggest that it is not only when a friend might incur an actual loss (e.g., in Powers et al. (2018) the friend could lose money) – even when the worst outcome is not gaining or losing anything, it was sufficient to elicit prosocial motives.

The upper limit of this age-related effect (22.1 years) was near the upper bound of the sample (22.8 years), and therefore should be interpreted with caution as the sample was sparser at the bounds. Future research could evaluate the reliability of this finding by collecting a wider range of ages. Alternatively, it could be that this effect diminishes completely into adulthood, which might reflect better strategizing among the oldest participants in this sample, whose earnings did not decrease in the Opposite condition as younger participants’ did.

The Identical condition did not induce significant change in risky choices compared to Baseline. Both the participant and their friend could receive what participant deemed the “better” option, so there were no competitive social motives to factor in. This interpretation is additionally supported by the fact that in the Identical condition, the original model outperformed the revised model (see Supplemental Materials), indicating the parameter that weighed in potential friend outcome as a competing source of utility did not improve fit to choices. Thus, in the Identical condition, the lack of tradeoff between maximizing one’s own gains and one’s friend’s presumably failed to elicit the prosocial motives observed in the Opposite condition. This highlights the specificity of early adolescent prosocial motives as being limited to circumstances in which there is an opportunity to feature prosocial gestures that are costly to oneself. This is in line with past work showing the strategic environment (e.g., social contexts), interacts with individual preferences, especially when decisions might impact players in an unequal fashion, because they might influence whether self-interest or prosocial considerations prevails (Fehr & Schmidt, 1999).

### Friend Observation

Friend observation induced an overall relative increase in risk acceptance but no age-related differences when there were identical outcomes for the dyad. Since Powers (2018) observed age-related changes with an in-person setup in a similar context, we speculate that the observation effect might have been diluted in our study due to dyads not being in the same room physically and no direct encouragement. One possibility is that observation over Zoom evoked a weaker sense of the friend’s presence, thereby alleviating the participants’ concerns for potentially awkward post-task interactions that might have occurred if the friend co-participated in lab and watched the participant assign them less favorable options. A meta-analysis showed that direct encouragement from the peer produced a larger effect size for observation effect compared to the subtle effect size in its absence (Powers et al., 2022). Future work evaluating peer influence on risk taking online should ascertain the influence of these hypothesized factors and consider emerging literature on how virtual platforms such as Zoom impact interpersonal interaction.

It is unclear what underlies the overall increase in risk acceptance caused by friend observation in the Identical context. It is possible that the potential excitement and arousal from having their friend as an audience boosted risk acceptance, as it has been shown that sharing an outcome equally with a friend elicited greater subjective rating of excitement and arousal than a non-friend (Fareri et al., 2012). Future research could incorporate physiological measures to explicitly test whether arousal bolsters risk acceptance when a friend who stands to win by one’s decision is observing, and whether that generalizes to other contexts.

There were also no overall or age-related changes caused by observation when the dyads received opposite outcomes. Observation also did not impact how much participants weighed friend outcome into their decision in this context, further supporting this inference. It is worth noting that participants knew their friend’s take-home pay would (partially) reflect the advantageousness of choices assigned to them by their friend. This may have been sufficient to evoke a latent sense of accountability irrespective of active observation.

### Limitations and Future Directions

Our analytical approach was data-driven and exploratory in nature with the aim to capture age-related shifts without imposing a presupposed shape. Instead, we treated age continuously and used tools sensitive to nonlinear patterns which sidesteps assumptions of linearity and definitional issues related to categorical treatment of data. We hope this approach will contribute to a detailed account of how risky decision making changes from early adolescence to early adulthood. However, this study is limited by investigating age-related changes in risky choices cross-sectionally. Future work should explore this behavior in a longitudinal sample. We also did not explore the degree of intimacy or stability of friendship between the dyads. Incorporating this variable into future work could enrich understanding of friend dynamics in further shaping the influence of peers on decisions about risk.

An additional consideration concerns our choice set, for which on average, the risky option was more economically advantageous. This type of choice set is commonly used to combat participants’ natural tendency for risk aversion. This might have limited our ability to examine risk in disadvantageous contexts. Future work could use a different set of choices varying the advantageousness of risks to further disentangle these concepts. Additionally, our choice set did not include risky choices in the loss domain, due to feasibility concerns for overly long study sessions and the potential for attrition over multiple sessions. The loss domain could provide crucial insight in how friend outcome might shape adolescents’ risky choices differently, however, and is an important avenue for future studies to examine.

### Conclusion

This study used analyses of continuous age and both computational and behavioral measures to chart the changes in risky decision making from early adolescence to young adulthood under different social-motivational contexts. We found that opposite outcomes for oneself and one’s friend elicited a robust shift in risk preferences in comparison to when risky choices only impacted oneself, and that younger adolescents are more sensitive to the influence of this social context, incurring more losses and disadvantageous risks. This suggests that overall, early adolescence is a period in which motivation is high to exhibit behaviors that showcase self-sacrifice that benefits peers, highlighting the potency of the social environment during this time of life.

## Acknowledgments

This work was partially supported by the Oscar M. Ruebhausen Fund at Yale Law School and Harvard University. We thank Alexandra Rodman for guidance on statistical methods, Juliet Davidow and Katherine Powers for modeling guidance, Erik Kastman for technical assistance, and Taylor Heffer, Hedy Kober, Camille Phaneuf, Elizabeth Phelps, and Gideon Yaffe for helpful discussion.

## Author Contributions

Leah H. Somerville and Laurel E. Kordyban designed the study. Laura Cegarra, Deanna A. Youssoufian, Melanie J. Grad-Freilich, Laurel E. Kordyban collected data. Yelina Yiyi Chen, Gail M. Rosenbaum, Haoxue Fan, and Leah H. Somerville conducted computational modeling analysis. Yelina Yiyi Chen, John C. Flournoy, Tianxiang Li, Patrick Mair, Leah H. Somerville conducted statistical analysis. Yelina Yiyi Chen, Gail M. Rosenbaum, Haoxue Fan, and Leah H. Somerville wrote and edited the manuscript.

## Data Availability Statement

The data that support the findings for this study are openly available at https://osf.io/9brsg/.

## Supplementary Material


