# Supplementary material for: Social Contexts Requiring Adjudication Self- and Peer-Interest Differentially Alter Risk Preferences Across Adolescence
Source: Open Mind (Camb). 2025 Apr 22;9:540–58. doi: 10.1162/opmi_a_00201 (PMC12058330; doi:10.1162/opmi_a_00201)
Supplement: Supplementary file 1 [file opmi-09-540-s001.docx]

Supplementary Materials

To support

Social contexts adjudicating self- and peer-interest differentially alter risk preferences across adolescence

Overview of contents

1. Supplementary Methods
2. Original expected utility (EU) model
3. Simulation and recovery
4. Preliminary model comparison: Expected utility versus prospect theory models
5. Decisions on model usability by condition
6. Model-based exclusions
7. Posterior predictive check
8. Revised model for Opposite context conditions
   1. Parameter recovery
   2. Model comparison with the revised vs. original model
9. Supplementary Results
   1. Results using proportion of risky choices as dependent variable
      1. Baseline risky choices
      2. Age-related shifts in risky choices evoked by Identical friend outcome
      3. Age-related shifts in risky choices evoked by Opposite friend outcome
      4. Age-related shifts in risky choices evoked by friend observation by context
   2. Distributions of actual and simulated random earnings
   3. Distributions of Alpha by condition
   4. Results of friend predicted Alpha
   5. Why the revised model is not used for conditions using the Identical context
10. Sensitivity Analyses
11. Baseline risky choices
12. Age-related shifts in risky choices evoked by friend outcome
13. Age-related shifts in risky choices evoked by friend observation in Identical and Opposite contexts
14. References
15. **Supplementary Methods**

**A. Original expected utility (EU) model**

$$\begin{aligned} EU=pv^{\alpha}\#original expected utility model \end{aligned}$$

1. Simulation and recovery

To verify that our parameter of interest (Alpha) is recoverable, we simulated choice data of 10,000 subjects each with 35 trials, with values of each parameter in the model drawn randomly and uniformly from the range of possible parameter values. We chose a range sufficient to explain the range of risk preferences that can be captured by our task for our parameters during simulation (0.02 < Alpha < 3.32, 1e-06 < Beta <10). In particular, we calculated the lower bound (0.035) for α by solving the following equation:

$${0.9\times100}^{\alpha}=5^{\alpha}$$

The reasoning is as follows: given our choice set, the most risk averse individual would prefer the safe option ($5 with certainty) to the risky option with the highest expected value in our choice set, which is winning $100 with 0.90 probability. Under the same logic, the most risk seeking individual would prefer the risky option with the lowest expected value in our choice set ($10 with 0.1 probability) to the certain option ($5). We therefore calculated the upper bound (3.32) for α by solving the following equation:

$${0.1\times10}^{\alpha}=5^{\alpha}$$

An individual who would not select the lowest EV risky option over the safe option but would select the second lowest ($10 with 0.25 probability) from our choice set would solve the following equation:

$${0.25\times10}^{\alpha}=5^{\alpha}$$

The resulting Alpha is 2. The choice set could have limited the model’s precision in distinguishing between Alpha values ranging from 2 to 3.32, as the difference between these two values lies in one choice.

We fit computational decision models at the subject level and used the mfit toolbox (Gershman, 2016) for MATLAB, which uses *fmincon* (*MATLAB 2018b; Mathworks*). For parameter recovery, we fit the simulated data using the abovementioned bounds for Beta (1e-06 < Beta <10) and slightly wider range for Alpha (0 < Alpha < 3.5). By slightly widening the search space, we are able to determine if a person’s Alpha is truly unrecoverable by seeing whether their value lies between 3.32 and 3.5; if we had capped fitted Alpha at 3.32 and the fitted Alpha is 3.32, we wouldn’t know whether that is really within the bounds or whether the model does not have the option to put it out of bounds.

We fitted the simulated data using the same EU model that generated it. Recoverability of model parameters is defined as the correlation between the parameter that generated the data and the parameter produced through model fitting (Wilson & Collins, 2019). We ran correlations between parameters used to simulate the data (“input parameters”) and parameters fit by *fmincon* (“fitted parameters”). Although Beta recovery was mediocre (*r* = .400; Alpha <= 2: *r* = .594; Alpha > 2: *r* = .057), recovery for Alpha was quite reliable (*r* = .922) with a drop after Alpha exceeds 2 (Alpha <= 2: *r* = .935; Alpha > 2: *r* = .049). As mentioned above, Alpha = 2 is the value at which one would be indifferent between the risky and safe options for the second riskiest choice in our choice set. The fact that values of Alpha were indistinguishable between 2 and 3.5, is in line with the notion that such values (mostly) do not yield different choices. Within this range, there was not a difference in recoverability above vs. below the Alpha necessary to explain the range of risk preferences in the task (3.32). Together, these results suggest that the model is limited in its ability to distinguish small differences among the most risk-seeking participants, likely due to the choice set. Reassuringly, input values of Alpha above 2 did not yield recovered values below 2, so participants who are in this maximally risk seeking range are nearly always identified as such based on their Alpha (except where Beta was very low, such that participants frequently do not choose the option with higher EU) (Figure S1).


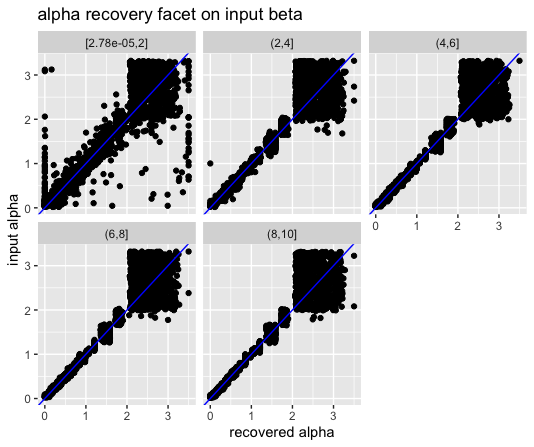


Figure S1. Recovery of Alpha based on different thresholds of input (simulated) Beta. *x*-axes denote recovered (fitted) Alpha. *y*-axes denote input (simulated) Alpha. The range on top of each panel denotes the range of input Beta that was used to generate the simulated data.

1. Preliminary model comparison: Expected utility versus prospect theory models

According to prospect theory and past research (Tversky & Kahneman, 1992; e.g., Gonzalez & Wu, 1999; Hsu et al., 2009; ) nonlinear weighting of probabilities is an important bias in decision making. Our choice set was designed according to an expected utility framework (Levy & Glimcher, 2011; Levy et al., 2012; Tymula et al., 2012) and not a prospect theory framework, and we did not expect that prospect theory based models would fit the data well. Nevertheless, for completeness and as a preliminary analysis step, we fit two additional models in the latter framework to determine whether incorporating a probability weighting function could improve the model fit. The first prospect theory model is constructed by combining the expected utility function and the following single-parameter weighting function:

$$\begin{aligned} w\left( p \right)= \frac{p^{\gamma}}{\left( p^{\gamma}+\left( 1-p \right)^{\gamma} \right)^{\frac{1}{\gamma}}}\#prospect theory model \end{aligned}$$

Therefore, this prospect theory model includes three free parameters: Alpha, Beta, and Gamma, where Gamma ($\gamma$) controls the weighting of probabilities ($p$). The second prospect theory model we fitted included only Beta and Gamma, i.e., assuming the decision-maker is risk neutral. We examined the parameter recoverability of the two prospect theory models before fitting them to real data using the aforementioned procedure. These correlations between input and fitted parameters are displayed in Table S1, indicating poor parameter recovery for the prospect theory models. We conclude that it is not feasible to incorporate prospect theory into our computational approach and which is likely due to limitations of the choice set.

Table S1. Parameter recoverability for expected utility and two prospect theory models

|  | Alpha, Beta (EU model) | Alpha, Beta, Gamma (prospect theory model) | Beta, Gamma (prospect theory model) |
| --- | --- | --- | --- |
| Alpha correlation (fitted, simulated input) | 0.922 (when Alpha<=2: 0.935; when Alpha>2: 0.049) | 0.206 (when Alpha<=2: 0.204; alpha > 2: 0.024) |  |
| Beta correlation (fitted, simulated input) | 0.400 (when Alpha<=2: 0.594; when Alpha>2: 0.057) | 0.138 (when Alpha<=2: 0.222; when Alpha>2: 0.006) | 0.52 |
| Gamma correlation (fitted, simulated input) |  | 0.003 (when Alpha<=2: 0.005; when Alpha> 2: -0.0004) | 0.028 |

1. Decisions on model usability by condition

We decided we would not use the original model if a given condition had more than 30 participants with a negative log likelihood (LL) that exceeded the value of negative LL for an agent who chose randomly for all 35 trials:

$$-ln \left( 0.5 \right) \times35=24.26$$

According to the criterion, which translates to an AIC of 52.52, we decided not to use the original model for the Opposite condition, Observed Opposite, and Unobserved Opposite conditions (Table S2).

Table S2. Number of unusable participants by condition

| Conditions | *N* with negative LL > 52.52 |
| --- | --- |
| Baseline | 0 |
| Friend Predicted | 0 |
| Opposite | 39 |
| Identical | 0 |
| Unobserved Opposite | 31 |
| Observed Opposite | 37 |
| Unobserved Identical | 4 |
| Observed Identical | 6 |

1. Model-based exclusions

The criteria described in the Methods - Model-based exclusion section resulted in the following exclusions (Table S3). We conducted sensitivity analyses including and excluding participants whose Alpha is theoretically possible but exceeds 2, due to simulations showing poor recovery of Alpha in this range. See Sensitivity Analyses.

Table S3. Model exclusions by condition

| Conditions | Number of participants excluded | Number of analyzed participants | percentage of participants excluded |
| --- | --- | --- | --- |
| Baseline | 1 | 127 | 0.78% |
| Opposite | 10 | 118 | 7.81% |
| Identical | 0 | 128 | 0.00% |
| Unobserved Identical | 12 | 111 | 9.76% |
| Observed Identical | 10 | 113 | 8.13% |
| Unobserved Opposite | 5 | 118 | 4.07% |
| Observed Opposite | 8 | 115 | 6.50% |

These exclusions apply to analyses of Alpha and Weight_friend_ as dependent variables only, as they were derived from computational models. No participant was excluded for analyses using simulated earnings or proportion of risky choices.

1. Posterior predictive check

We ran a posterior predictive check on the Expected Utility model. For each real participant in each condition, we simulated 100 participants with the same parameter estimates. As a model-free measure, we calculated that participant’s proportion of risky choices and the proportion of risky choices across the corresponding 100 simulated participants for a given condition. The correlation between the proportion of risky choices for each real and simulated participant was high for all conditions (*r* > .8).

Table S4. Results of posterior predictive check

| Conditions | Correlation between real and simulated choices |
| --- | --- |
| Baseline | *r*=.98 |
| Friend Predicted | *r*=.98 |
| Identical | *r*=.98 |
| Unobserved Identical | *r*=.84 |
| Observed Identical | *r*=.83 |

**B. Revised model for Opposite context conditions**

$$\begin{aligned} EU=\left( 1-w \right)p_{self}{v_{self}}^{\alpha}+w\left( p_{friend}{v_{friend}}^{\alpha_{friend}} \right)\#revised model \end{aligned}$$

Through the same process outlined for our original model, we determined that our revised model was recoverable in its parameter of interest within the following bounds: Alpha ([0,2]).

1. Parameter recovery

Table S5. Parameter recovery for revised model.

|  | Weight_friend_ | Alpha |
| --- | --- | --- |
| Correlation between simulated input and fitted value | *r*=.73 | *r*=.78 |

Beta (*r* =.35) is not a parameter of interest but rather a nuisance parameter that is meant to absorb noise in the data. Generally, the fit of noise parameters is not germane unless the overall model fit is poor, which we have shown to be *not* true in our case. Otherwise, model recovery focuses on parameters carried forward for inference, which in our case, is Alpha and Weight_friend_, and both recovered well (*r* >.7).

As Ballard & McClure (2019) presented in detail, the tradeoff between parameters of interest (i.e., the underlying psychological process we are interested in) and decision noise is a general problem for models that attempt to describe noisy psychological processes. Low correlations between decision noise and parameters of interest provide evidence indicating that there is little trade-off of parameters against one another, bolstering confidence that they are individually identifiable (Wilson & Collins, 2019). In our study, correlations between fitted values of Beta and Weight_friend_ (*r* = .09) and Beta and Alpha (*r* = *-.*03) are extremely low. The fact that Alpha / Weight_friend_ and Beta are not highly correlated (i.e., they are identifiable), paired with the fact that Alpha (the parameter of interest) is recoverable, builds confidence that the Alpha parameter is indeed robust and suitable for inference.

1. Model Recovery with the revised vs. the original model

Like the process outlined for the original model, we simulated data using the original and revised model respectively, and fitted the resulting simulated data with both models respectively. Our new model outperformed the original model for the following bounds for Weight_friend_ [1e-06,1].

Table S6. Given what the generating model is (row), percentage of times that AIC suggests (column) is a better model.

| Fit model  Data generating model | Original | Revised |
| --- | --- | --- |
| original | 88.7% | 11.3% |
| revised | 37.7% | 62.3% |

Additionally, we explored the possibility that individuals use their own alpha as a proxy of their friend’s risk preference as well. To this end, we compared the median AIC resulting from using Alpha*_friend_* vs. participant’s own Alpha for the second term in our revised model. Using Alpha*_friend_* resulted in a better fit of the data (median AIC using Alpha*_friend_* vs. one’s own Alpha: 30.056 vs. 30.593), indicating that using an estimated friend preferences better fit individual’s’ choices in the Opposite context, compared to using their own risk preference as a proxy for their friend’s preference. This means that participants tended to represent their friend’s risk preferences as distinct from their own.

1. **Supplementary Results**
2. **Results using proportion of risky choices as dependent variable**
3. Baseline Risky Choices


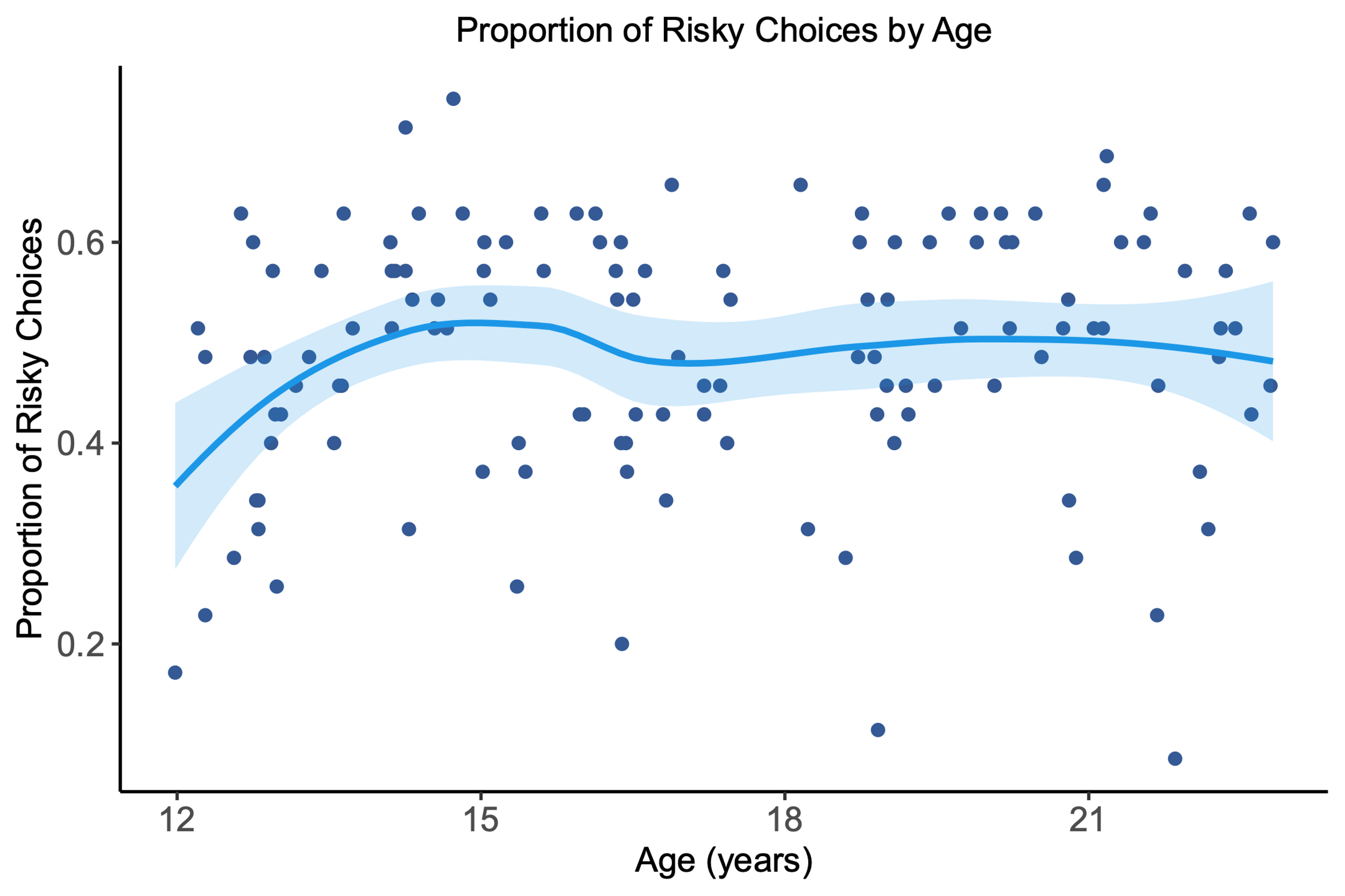

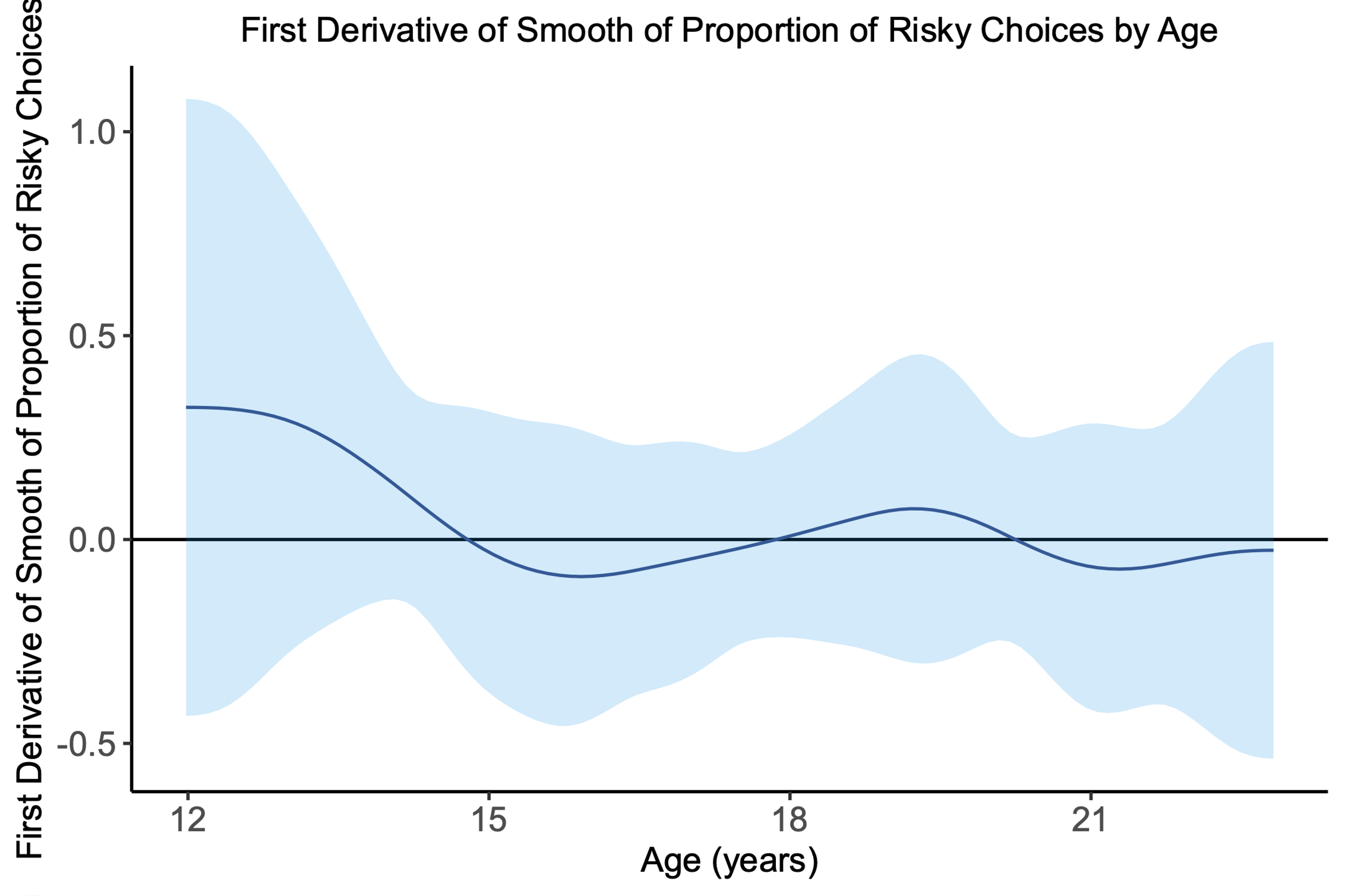


A.

B.

Figure S2. Age-related patterns of risk seeking (proportion of risky choices). (A) Scatter plot of raw data. *x-*axis denotes age in years. *y*-axis indicates proportion of risky choices (number of risky choices divided by total number of trials in a condition). The blue line is a smoothing line over the data points to visualize age trend in the data. Shaded regions represent 95% CIs around the trend line. (B) First derivative of the GAM model. Shaded area represents 95% simultaneous CIs.

This analysis evaluated the relationship between proportion of risky choices and age in the Baseline condition. On average, participants chose risky choices 49% for the time (*SD*=0.13). The simultaneous CI of the first derivative of the spline included 0 at all ages (Fig. S2B). This indicates that in the age range of our sample, participants’ risk aversion remained constant as age increased.

1. Age-related shifts in risky choices evoked by identical friend outcome

Comparing the Identical condition to Baseline, we examined whether joint outcomes (risky choice identically impacting oneself and one’s friend) influence one’s baseline risk preferences. There were no differences between the Identical condition and Baseline overall, or age-related changes on the proportion of risky choices (Mean of Identical vs. Baseline: .51 vs. .49; *B*=0.05, *t*=0.99, *SE*=0.05, *p*=.323). This indicates that individuals did not proportionally take a different amount of risks when the choice stood to benefit oneself only, or oneself and one’s friend.

1. Age-related shifts in risky choices evoked by opposite friend outcome


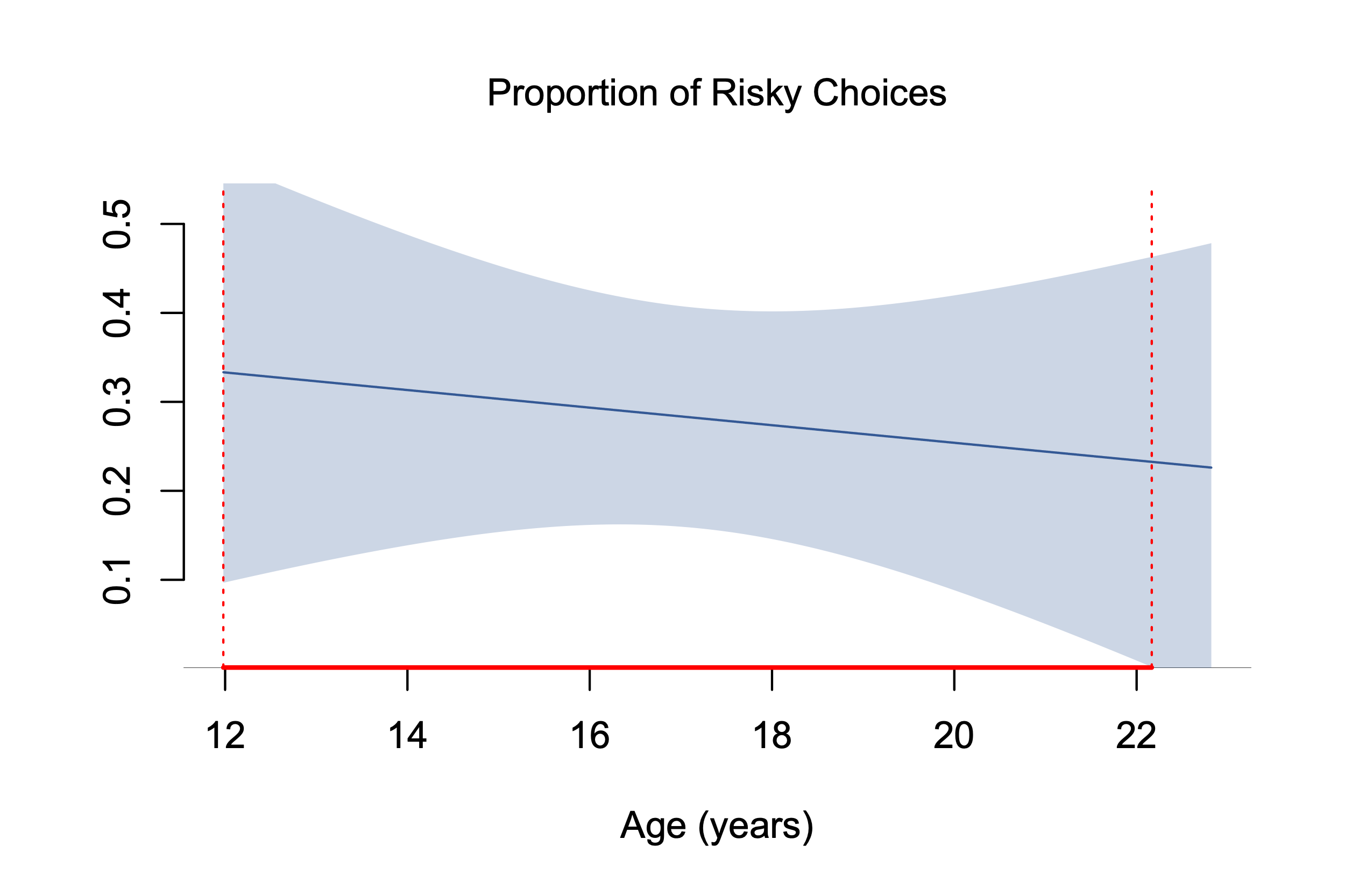


Figure S3. Age-related patterns of proportion of risky choices: difference of fit estimates of the GAM model. Positive values indicate greater proportion of risky choices at Opposite than Baseline, negative values indicate the reverse, and 0 indicates proportions of risky choices are not significantly different across the conditions. Shaded area represents 95% simultaneous CIs of the difference between fit estimates at each age points. Dotted red lines show that between ages 12.0-22.2 years, the 95% simultaneous CIs do not include 0, indicating significant age-related changes.

In the Opposite condition, participants’ choices caused their friends to receive their unchosen option. Analyses determined the extent to which participants changed the proportion of risky options they chose compared to Baseline when the decision only concerned themselves. On average, participants chose risky options more often for themselves when it meant that their friend would otherwise have to accept the risky option (Mean of Opposite vs. Baseline: .56 vs. .49; *B*=0.28, *t*=5.50, *SE*=0.05, *p*<.001). This trend was significant in ages 12.0 to 22.2 years (Figure S3*)*. Thus, participants from almost the entire age range of our sample chose proportionally more risky options when they had to decide whether to keep their preferred option or assign it to their friend instead.

1. Age-related shifts in risky choices evoked by friend observation by context

In the Identical context, participants chose proportionally more risky options when their friends watched them make decisions that yielded the same outcomes for them both, compared to when unwatched (Mean of Observed Identical vs. Unobserved Identical: .71 vs. .69; *B*= 0.13, *t*=2.47, *SE*= 0.05, *p*=.014).

In the Opposite context, there were no significant differences by observation (Mean of Observed Opposite vs. Unobserved Opposite: .55 vs. .55; *B*=0.006, *t*=0.13, *SE*=0.05, *p*=0.900).

There were no significant age-related differences by observation in either Identical or Opposite contexts.

**B. Distributions of actual and simulated random earnings**


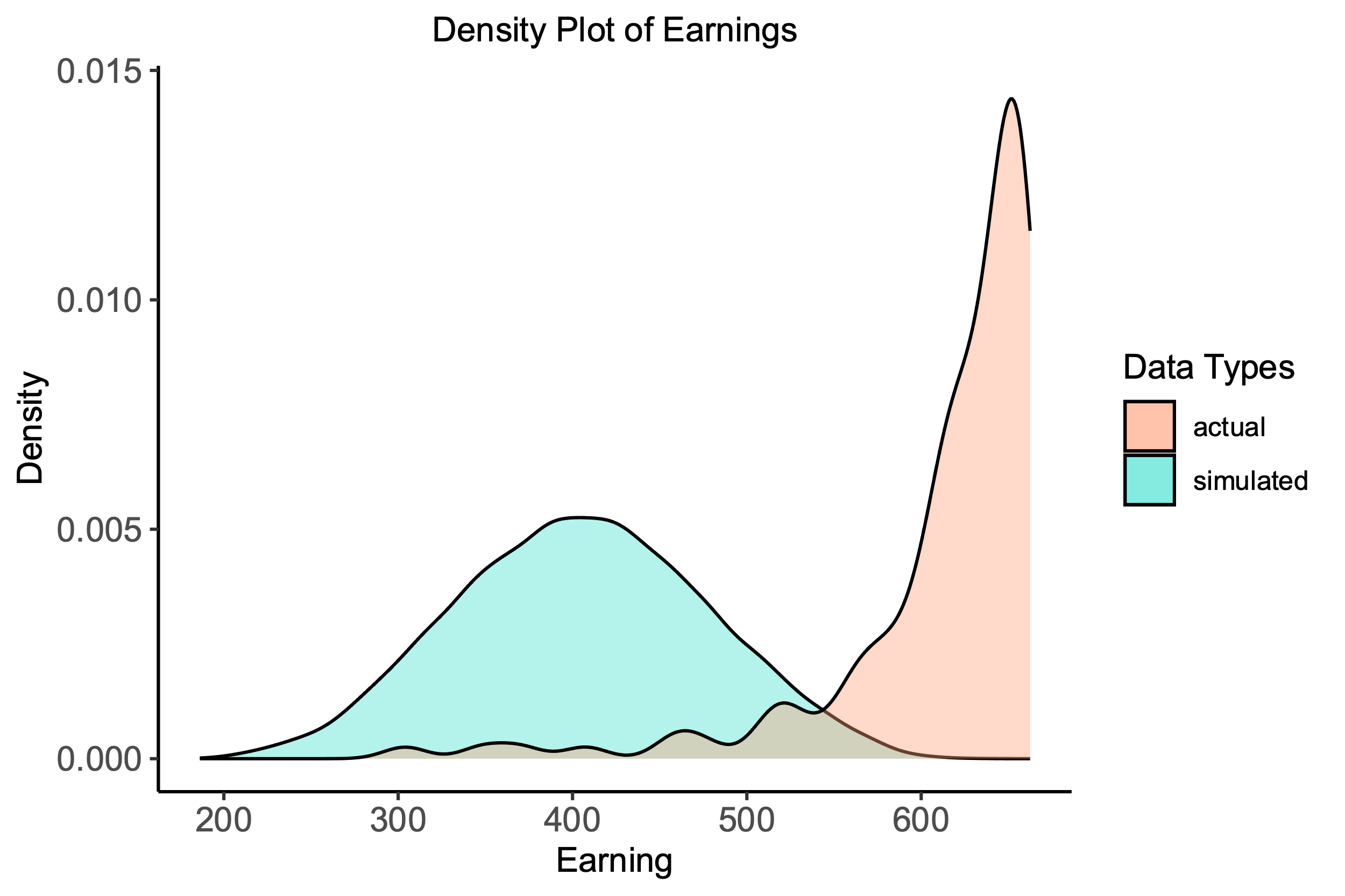


Figure S4. Distributions of actual and simulated random earnings: the blue area represents simulated earnings, and the red area represents actual data. We simulated 10,000 participants choosing at chance (50%) with 35 trials each in the Baseline condition, and compared this distribution to that of the actual data from the Baseline condition (Mean of simulated random earnings vs. actual data: $406.06 vs. $614.52; Standard deviation of simulated random earnings vs. actual data: 72.14 vs. 63.75).

**C.**  **Distributions of Alpha values by condition**

**
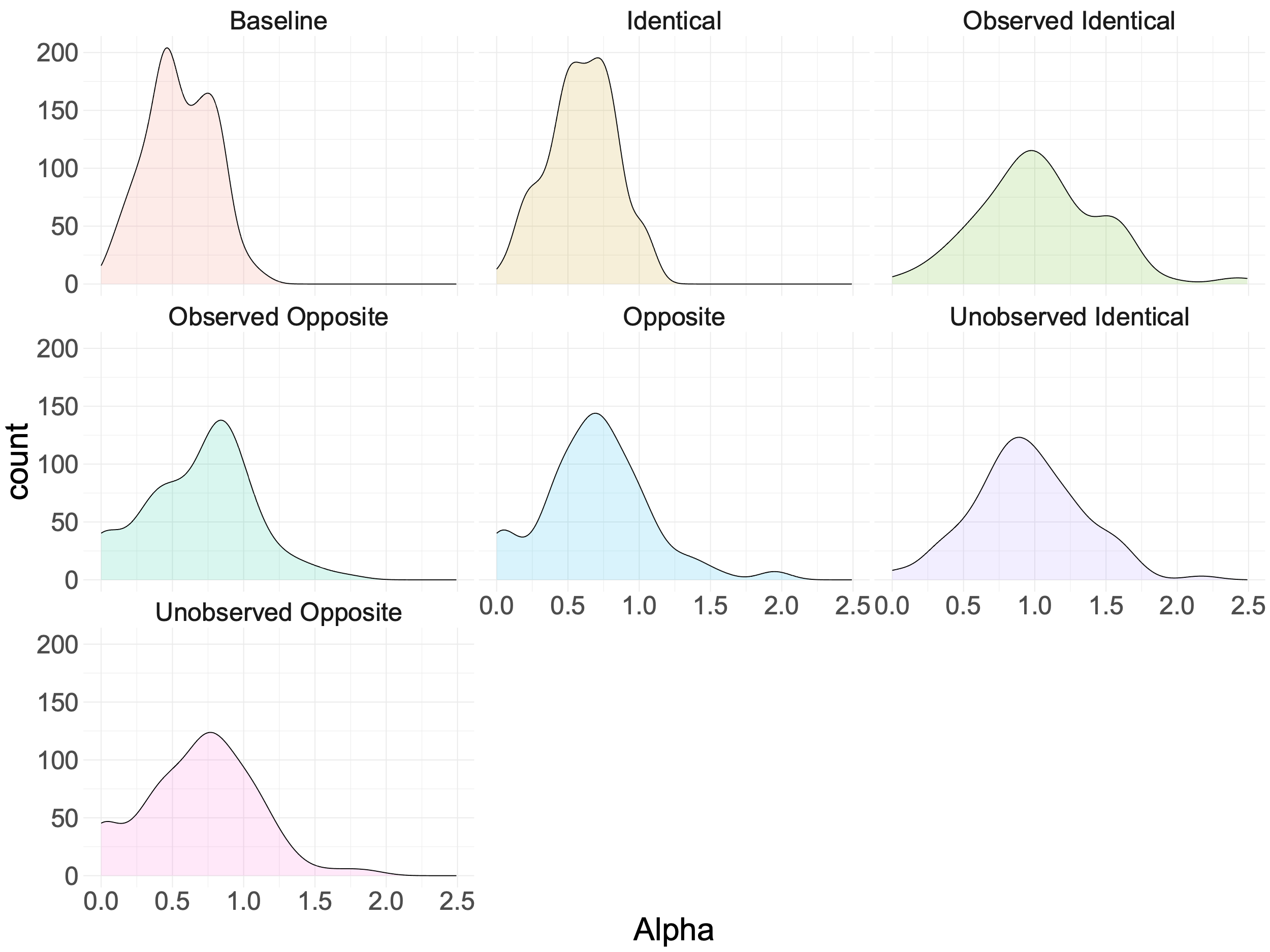
**

Figure S5. Distributions of Alpha in each condition of the study. *x*-axis indicates Alpha value, *y*-axis (count) indicates the frequency of Alpha taking on a specific value.

**D. Results of friend predicted Alpha**

We evaluated the degree of consistency between participants’ own risk preferences and what they envisioned the risky choices to be if their friend was choosing for themselves (i.e., the Friend Predicted condition). By comparing the Friend Predicted condition to participants’ own Baseline condition, we can evaluate whether participants thought their friend’s choice would be the same, or riskier, than their own. This analysis follows the same logic as section “Treatment of age” paragraph 2 in the main paper.

The overall mean risk preference (Alpha) derived from participants’ predicted choices for their friend is 0.63 (SD = 0.34) and does not change with age, as demonstrated by the fact that the first derivative of the spline for Predicted Friend Alpha includes 0 at all ages. We used a factor smooth interaction described in Analysis section “Treatment of age” paragraph 3 of main text to ascertain whether there are age-related changes between one’s own Baseline Alpha (for solo decisions) vs. their predicted Friend Alpha (their prediction of their friend’s solo decisions). There was a main effect of Condition (Mean of Baseline vs. Predicted Friend Alpha: 0.55 vs. 0.63, B=-0.08, *SE*=0.03, *t*=-2.96, *p*=.003), indicating that on average, participants predicted their friend to be relatively more risk seeking than they themselves are. There are no age-related changes, indicated by the fact that the Simultaneous CIs included 0 for all ages. In all, participants across the age range consistently thought their peer was more risk seeking than they themselves were.

**E. Why the revised model is not used for conditions using the Identical context**

Although our revised model was designed with the Opposite context in mind, we checked whether we should consider using it for the Identical, Observed Identical, and Unobserved Identical condition, since they involved friend outcome in the decision as well, though in a different dynamic. Across all three Identical context conditions, the median AIC of the original model is smaller than that of the revised model, suggesting that the original model, which did not treat friend outcome as a competing source of utility to one’s own, provides a better model fit for these conditions (Table S7).

Table S7. Model comparison for Identical conditions

| Condition | Median AIC, Original Model | Median AIC, Revised Model |
| --- | --- | --- |
| Identical | 15.159 | 17.159 |
| Observed Identical | 27.325 | 28.966 |
| Unobserved Identical | 25.278 | 26.687 |

1. **Sensitivity Analysis**

Our primary analysis excluded participants based on the model-based exclusion section in the main text. As pre-registered, we conducted two sets of sensitivity analyses using different exclusion criteria on Alpha fitted by the original model, which was used for the Baseline condition and all Identical context conditions. The first set of analyses exclude participants with fitted Alpha > 2, i.e., excluding participants whose Alpha is theoretically possible but exceeds 2, due to simulations showing poor recovery of Alpha within the range of 2-3.32 despite the recovery over the entire range of Alpha being robust. The second set of analysis had no exclusions, i.e., all participants’ data were used, including those with a theoretically impossible Alpha value based on our choice set (Alpha > 3.32). We did not conduct similar sensitivity analyses for Alpha fitted by the revised model by including data excluded in the primary analysis because when Alpha was not recoverable outside of the range [0,2] (see section I.B). In contrast, Alpha in the original model was still recoverable over the whole range used for recovery [0,3.5], though Alpha exceeding 3.32 was theoretically implausible given our choice set (see section I.A.1).

1. **Baseline risky choices**

Table S8. Number of participants in the Baseline condition under different exclusion criteria based on Alpha

| Exclusion based on Alpha | Number of participants in Baseline |
| --- | --- |
| No exclusions | 128 |
| *Exclude Alpha > 3.32 (Primary Analysis)* | *127* |
| Exclude Alpha > 2 | 127 |

Excluding Alpha > 2 resulted in the same usable data as the analysis reported in the main text (Table S8), see the corresponding section in the Results.

No exclusions: we fit our statistical model with no exclusions based on Alpha. The simultaneous CI of the first derivative of the spline included 0 at all ages (Figure S6B), which is the same as the primary finding that in the age range of our sample, participants’ risk aversion remained constant as age increased.

A.

B.


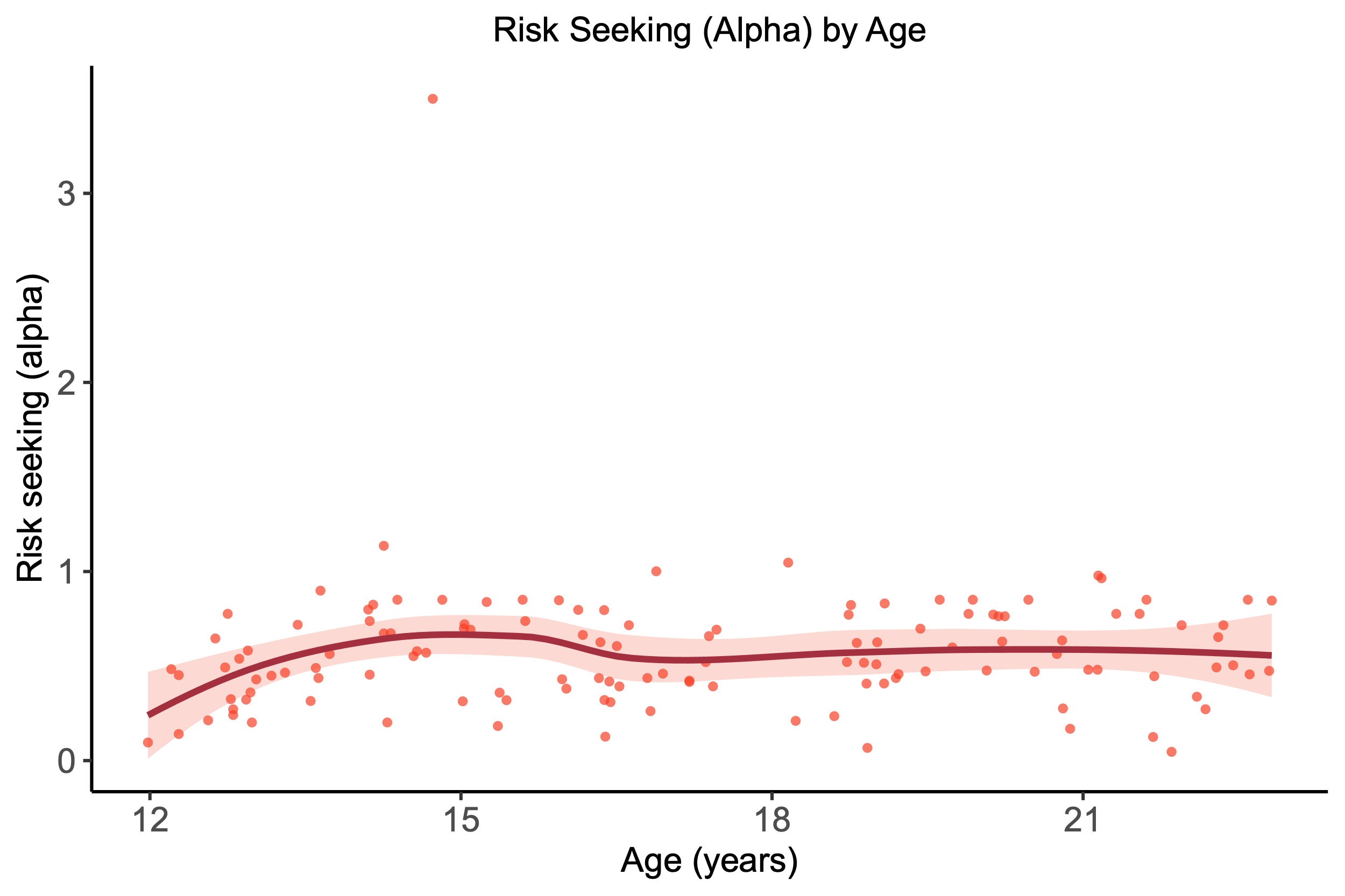

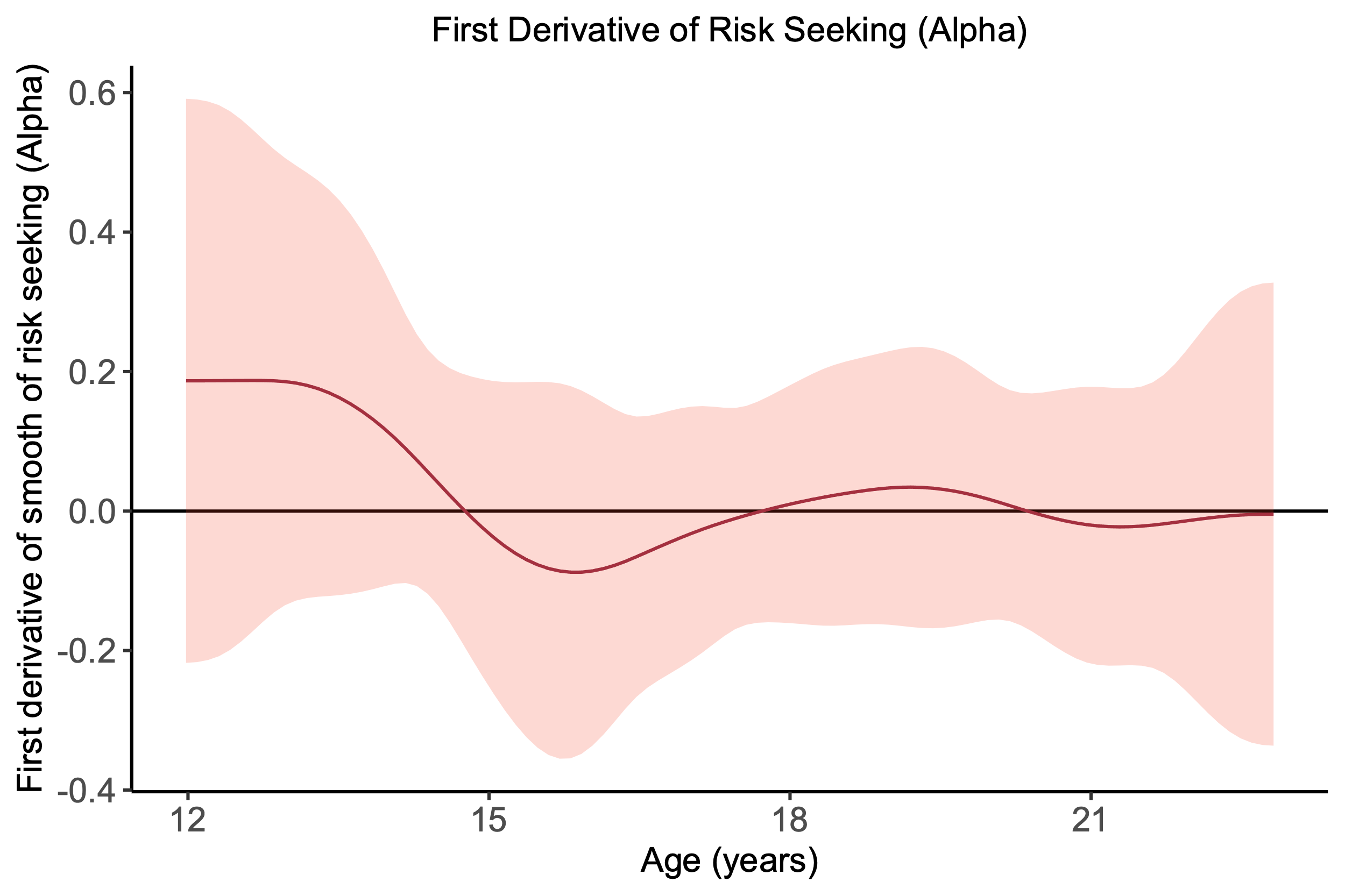


Figure S6. Age-related patterns of risk seeking (Alpha) with no exclusions. A. Scatterplot of raw data (Alpha values). The red line is a smoothing line over the data points to visualize the age trend in the data. Shaded region represents 95% simultaneous CIs around the trend line. B. First derivative of the GAM model. Shaded area represents 95% simultaneous CIs.

1. **Age-related shifts in risky choices evoked by friend outcome**

Table S9. Number of participants for Baseline and Identical conditions under different exclusion criteria based on Alpha.

| Exclusion based on Alpha | Number of participants in Baseline | Number of participants in Identical |
| --- | --- | --- |
| No exclusion | 128 | 128 |
| *Exclude Alpha > 3.32 (Primary Analysis)* | *127* | *128* |
| Exclude Alpha > 2 | 127 | 128 |

Excluding Alpha > 2, similar to the section above, resulted in the same usable data as the analysis reported in the main text (Table S9), see corresponding section in Results.

No exclusions:

1. *Identical condition vs. Baseline*

There was no significant difference between Baseline and Identical condition overall or age-related differences in (Mean of Identical vs. Baseline: 0.59 vs. 0.57; *B* = 0.02, *t* = 0.61, *SE* = 0.03, *p* = .543), which is similar to the main text (Mean of Identical vs. Baseline: 0.59 vs. 0.55; *B* = 0.04, *t* = 1.61, *SE* = 0.03, *p* = .108). This analysis confirms that the exclusion criteria did not have an undue influence on the primary findings, which is that individuals judge risk similarly when it stands to benefit oneself only, and oneself and one’s friend.

1. *Opposite condition vs. Baseline*

Overall, participants were more risk seeking for themselves in the Opposite condition (Mean of Opposite vs. Baseline: 0.69 vs. 0.57; *B* = 0.12, *t* = 3.53, *SE* = 0.03, *p* < .001), leading to the same inference as the primary analysis (Mean of Opposite vs. Baseline: 0.69 vs. 0.55; *B* = 0.14, *t* = 5.25, *SE* = 0.03, *p* <. 001). Unlike our primary analysis where we found significant age-related differences between age 12.3-15.1 years, we only found significant age-related difference between age 13.7-14.1 years with no exclusions (Figure S7). However, visualizing the raw data (Figure S8) revealed that the one participant that was excluded in the primary analysis but included here is an outlier with a theoretically impossible Alpha. Therefore, this result does not warrant alterations to our primary inferences drawn from our primary analysis.


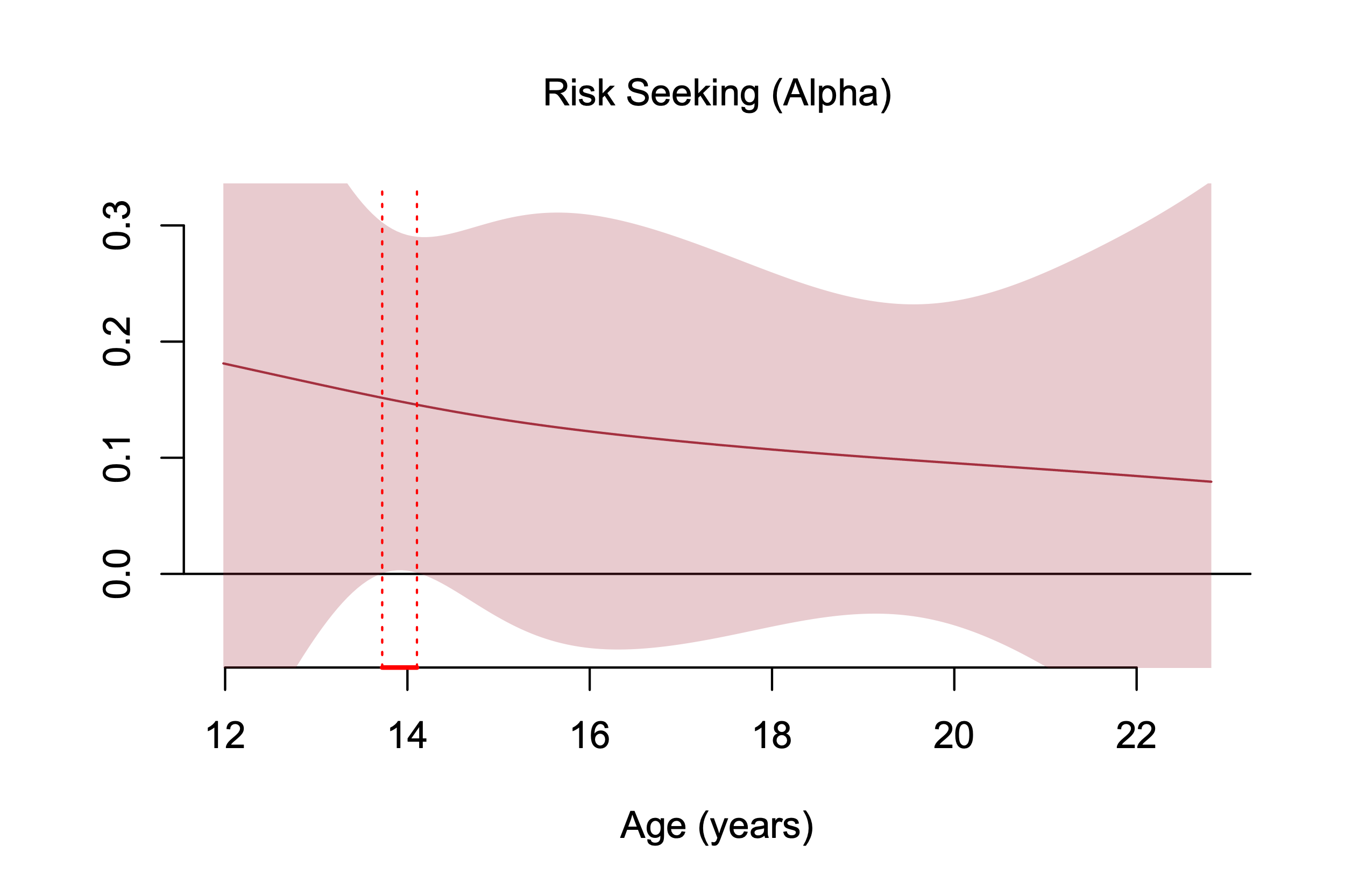


Figure S7. Age-related patterns of risk seeking: Difference of fit estimates of the GAM model. Positive values indicate risk seeking is greater at Opposite than Baseline, negative values indicate the reverse, and 0 indicates risk seeking is equivalent across the conditions. Shaded area represents 95% simultaneous Cis. Dotted red lines show that between ages 13.7-14.1 years, the 95% simultaneous CIs do not include 0, indicating significant age-related changes.

A.

B.


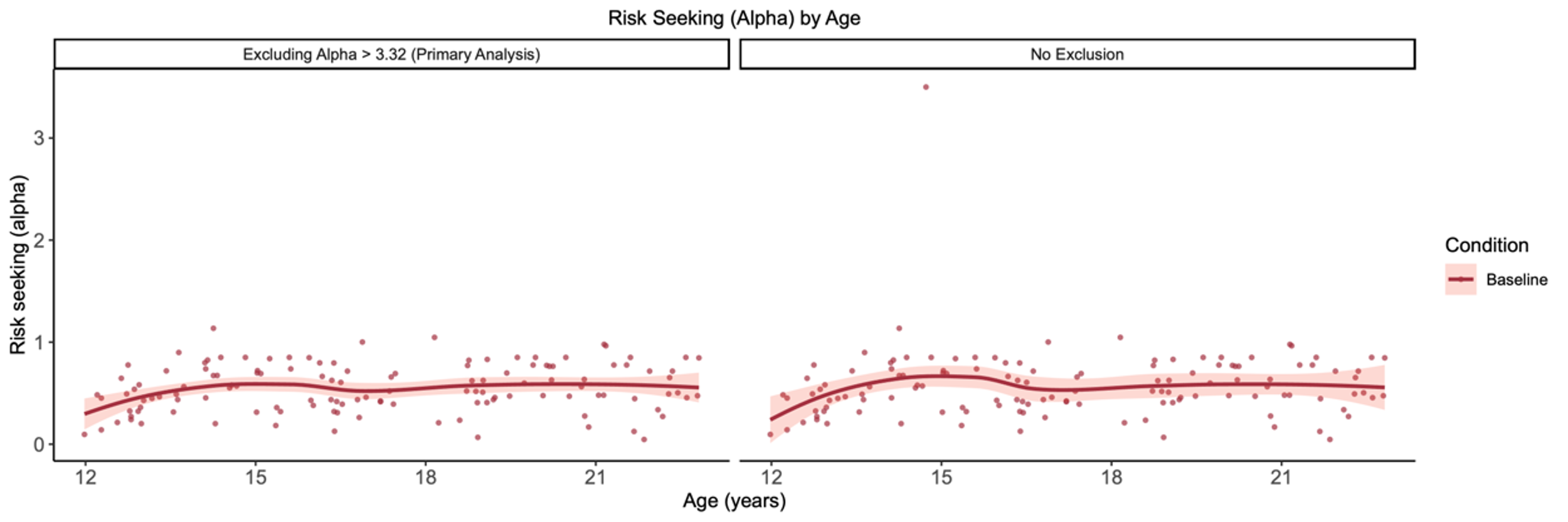

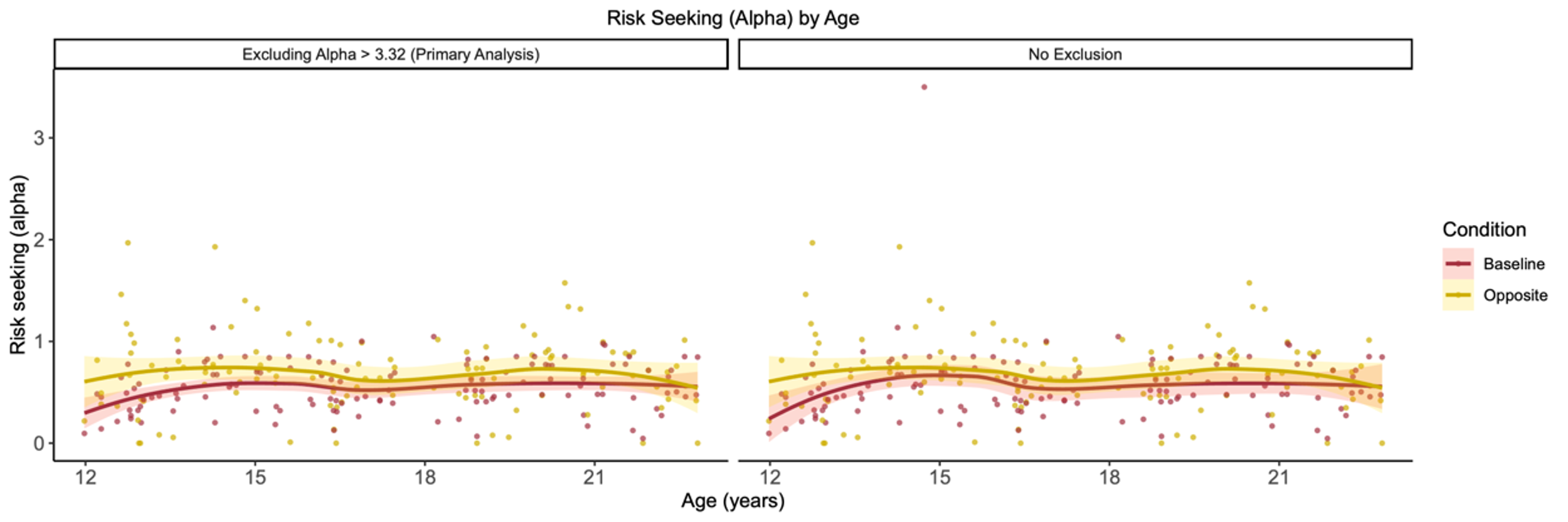


Figure S8. (A) scatterplot of raw data in Baseline in primary analysis vs. with no exclusion. (B) scatterplot of raw data in Baseline and Opposite in primary analysis vs. with no exclusion.

1. **Age-Related shifts in risky choices evoked by friend observation in Identical and Opposite contexts**

Table S10. Number of participants for Observed and Unobserved Identical conditions under different exclusion criteria based on Alpha.

| Exclusion based on Alpha | Identical | |
| --- | --- | --- |
|  | Observed | Unobserved |
| No exclusion | 114 | 114 |
| *Exclude Alpha > 3.32 (Primary Analysis)* | *113* | *111* |
| Exclude Alpha > 2 | 111 | 110 |

Because all three exclusion criteria resulted in different datasets (Table S10), we fit our statistical models with two preregistered thresholds for model-based exclusion on Alpha: one with no exclusion, and the other excluding participants with Alpha > 2.

1. *No exclusions*

Unlike our primary analysis where we found participants more risk seeking in the Observed Identical condition than in the Unobserved Identical condition (Mean of Observed Identical vs. Unobserved Identical: 1.04 vs. 0.94; *B* = 0.10, *t* = 2.65, *SE* = 0.04, *p* = .008), we did not find this effect with no exclusions (Mean of Observed Identical vs. Unobserved Identical: 1.06 vs. 1.01; *B* = 0.05, *t* = 0.97, *SE* = 0.05, *p* = .331). However, visualizing the raw data (Figure S9) revealed that the 4 participants that were excluded in the primary analysis but included here are outliers with theoretically impossible values at the upper bound of the aforementioned slightly widened range for Alpha recovery, making these data points unreliable. Therefore, this result does not warrant alternations for our primary inferences drawn from our primary analysis.


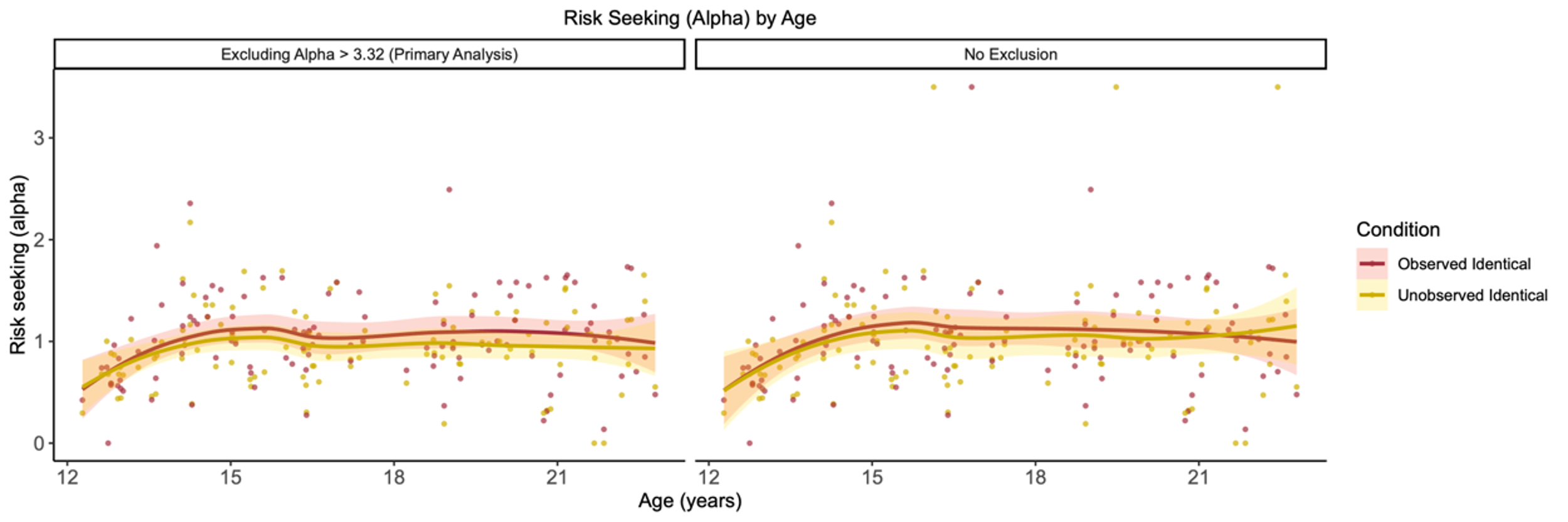


Figure S9. Scatterplot of raw data in Observed Identical and Unobserved Identical in primary analysis vs. with no exclusions.

There was no difference between Observed Opposite condition and Unobserved Opposite condition (Mean of Observed Opposite vs. Unobserved Opposite: 0.69 vs. 0.69; *B* = -0.001, *t* = -0.023, *SE* = 0.05, *p* = .982), leading to the same inference as the primary analysis (Mean of Observed Opposite vs. Unobserved Opposite: 0.69 vs. 0.69; *B* = -0.001, *t* = -0.04, *SE* = 0.04, *p* = .971). There were no age-related differences between Observed and Unobserved in both Identical and Opposite conditions, as was the case in the primary analysis.

1. *Excluding Alpha > 2*

Overall, participants were more risk seeking in the Observed Identical condition than in the Unobserved Identical condition (Mean of Observed Identical vs. Unobserved Identical: 1.01 vs. 0.93; *B* = 0.08, *t* = 2.31, *SE* = 0.03, *p* = .022), leading to the same inference as the primary analysis (Mean of Observed Identical vs. Unobserved Identical: 1.04 vs. 0.94; *B* = 0.10, *t* = 2.65, *SE* = 0.04, *p* = .008). There was no difference between Observed Opposite condition and Unobserved Opposite condition (Mean of Observed Opposite vs. Unobserved Opposite: 0.69 vs. 0.69; *B* = 0.002, *t* = 0.062, *SE* = 0.03, *p* = .951), leading to the same inference as the primary analysis (Mean of Observed Opposite vs. Unobserved Opposite: 0.69 vs. 0.69; *B* = -0.001, *t* = -0.04, *SE* = 0.04, *p* = .971). There were no age-related differences between Observed and Unobserved in both Identical and Opposite conditions, as was the case in the primary analysis.

**Conclusion**

These sensitivity analyses confirm that our exclusion criteria did not have undue influence on the primary findings.

1. **References**

Ballard, I. C., & McClure, S. M. (2019). Joint modeling of reaction times and choice improves parameter identifiability in reinforcement learning models. *Journal of Neuroscience Methods*, *317*, 37–44. https://doi.org/10.1016/j.jneumeth.2019.01.006

Gershman, S. J. (2016). Empirical priors for reinforcement learning models. *Journal of Mathematical Psychology*, *71*, 1–6. https://doi.org/10.1016/j.jmp.2016.01.006

Gonzalez, R., & Wu, G. (1999). On the Shape of the Probability Weighting Function. *Cognitive Psychology*, *38*(1), 129–166. https://doi.org/10.1006/cogp.1998.0710

Hsu, M., Krajbich, I., Zhao, C., & Camerer, C. F. (2009). Neural Response to Reward Anticipation under Risk Is Nonlinear in Probabilities. *The Journal of Neuroscience*, *29*(7), 2231–2237. https://doi.org/10.1523/JNEUROSCI.5296-08.2009

Levy, D. J., & Glimcher, P. W. (2011). Comparing Apples and Oranges: Using Reward-Specific and Reward-General Subjective Value Representation in the Brain. *Journal of Neuroscience*, *31*(41), 14693–14707. https://doi.org/10.1523/JNEUROSCI.2218-11.2011

Levy, I., Rosenberg Belmaker, L., Manson, K., Tymula, A., & Glimcher, P. W. (2012). Measuring the Subjective Value of Risky and Ambiguous Options using Experimental Economics and Functional MRI Methods. *Journal of Visualized Experiments : JoVE*, *67*, 3724. https://doi.org/10.3791/3724

Tversky, A., & Kahneman, D. (1992). Advances in prospect theory: Cumulative representation of uncertainty. *Journal of Risk and Uncertainty*, *5*(4), 297–323. https://doi.org/10.1007/BF00122574

Tymula, A., Belmaker, L. A. R., Roy, A. K., Ruderman, L., Manson, K., Glimcher, P. W., & Levy, I. (2012). Adolescents’ risk-taking behavior is driven by tolerance to ambiguity. *Proceedings of the National Academy of Sciences*, *109*(42), 17135–17140. https://doi.org/10.1073/pnas.1207144109

Wilson, R. C., & Collins, A. G. (2019). Ten simple rules for the computational modeling of behavioral data. *eLife*, *8*, e49547. https://doi.org/10.7554/eLife.49547
